# Lipopolysaccharide enters the rat brain by a lipoprotein-mediated transport mechanism in physiological conditions

**DOI:** 10.1038/s41598-017-13302-6

**Published:** 2017-10-13

**Authors:** Alejandra Vargas-Caraveo, Aline Sayd, Sandra R. Maus, Javier R. Caso, José L. M. Madrigal, Borja García-Bueno, Juan C. Leza

**Affiliations:** 1 Department of Pharmacology, Faculty of Medicine, Hospital 12 de Octubre Imas12, IUINQ, University Complutense, Madrid, 28040 Spain; 2 Escuela Nacional de Medicina y Homeopatía, Instituto Politécnico Nacional, Mexico city, 07320 Mexico; 3 Centro de Investigación Biomédica en Red de Salud Mental, CIBERSAM, Spain

## Abstract

Physiologically, lipopolysaccharide (LPS) is present in the bloodstream and can be bound to several proteins for its transport (i.e.) LPS binding protein (LBP) and plasma lipoproteins). LPS receptors CD14 and TLR-4 are constitutively expressed in the Central Nervous System (CNS). To our knowledge, LPS infiltration in CNS has not been clearly demonstrated. A naturalistic experiment with healthy rats was performed to investigate whether LPS is present with its receptors in brain. Immunofluorescences showed that lipid A and core LPS were present in circumventricular organs, choroid plexus, meningeal cells, astrocytes, tanycytes and endothelial cells. Co-localization of LPS regions with CD14/TLR-4 was found. The role of lipoprotein receptors (SR-BI, ApoER2 and LDLr) in the brain as targets for a LPS transport mechanism by plasma apolipoproteins (i.e. ApoAI) was studied. Co-localization of LPS regions with these lipoproteins markers was observed. Our results suggest that LPS infiltrates in the brain in physiological conditions, possibly, through a lipoprotein transport mechanism, and it is bound to its receptors in blood-brain interfaces.

## Introduction

LPS molecules are essential outer membrane components of most Gram-negative bacteria. These molecules are composed of three distinct domains: the O-specific polysaccharide or O-antigen, the core oligosaccharide and the hydrophobic moiety named lipid A^1^. Lipid A is a glucosamine-based saccharolipid, formed in most of the cases by two linked 2-amino-2-deoxy-d-glucopyranose (d-GlcpN) residues, phosphate groups and acylated with 3-hydroxy fatty acids at positions 2 and 3 of both GlcpN^1,2^. The acyl chain region of the lipid A moiety from LPS is recognized by CD14 and TLR-4/MD-2 receptors in most cells, triggering the innate immune signaling pathway, inducing NFκB nuclear translocation and in consequence the release of pro-inflammatory cytokines and the synthesis of some inducible inflammatory and oxido/nitrosative enzymes. This way, the Lipid A structure is related to its endotoxic properties, but some differences in potency have been described, depending on the microbial species, environmental conditions such as temperature^1–3^ and chemical modifications by adding or removing acyl chains and phosphate groups^3^. The hexa-acylated and phosphorylated lipid A from *Escherichia coli* (*E. coli*) is the strongest Toll-like receptor 4 (TLR-4) ligand, leading to highly efficient synthesis of proinflammatory cytokines^3,4^. In the absence of infection, levels of LPS in the blood stream are mostly attributed to increased intestinal permeability that can be produced or exacerbated by different conditions like a high-fat diet^5^, physical exercise^6^ and stress^7^. Pathologies, including atherosclerosis, alcoholism, obesity, chronic fatigue, schizophrenia, depression and others have been also associated with inflammation development induced by LPS as a product of intestinal permeability^8^. However, in physiological conditions, LPS blood levels have been detected^9–11^. In people in contact with livestock or animal by-products, an increase in LPS blood levels has been described as a protective mechanism for maintaining immune defenses^12^.

After the translocation of LPS-containing bacteria from the intestinal lumen to the bloodstream, the incorporation of LPS into micelles can occur, being then aggregated into chylomicron particles^5^. But LPS molecules may be also found in blood associated with plasma proteins such as LBP (LPS binding protein), soluble CD14 or incorporated in lipoproteins through binding to apolipoproteins (Apo). Specifically, the role of LBP has been mostly related to beneficial effects because it promotes LPS clearance from blood by transport to the liver and bile^13^. Also, ApoAI and ApoE, present in High-density lipoprotein (HDL) and ApoB, present in Low Density Lipoprotein (LDL), have been described to bind with high affinity to LPS^14–17^. Part of LPS clearance by the liver is through lipoprotein particle uptake carried out by several receptors including scavenger receptor class B type I (SR-BI) and LDL family receptors expressed in the liver^13^.

In recent years, some neuropsychiatric diseases such as depression and schizophrenia and related conditions such as chronic fatigue syndrome have been related to the increase of plasma LPS levels caused by *leaky gut* syndrome^18,19^ and the activation of the TLR-4 signaling pathway in the brain^20^. However, the infiltration of LPS into brain tissue has not yet been proved^21,22^, and it has not yet been clarified how peripheral LPS can stimulate the activation of the innate immune response in the brain because of the presence of the blood-brain-barrier (BBB)^23^. Structures lacking the BBB such as the circumventricular organs (CVOs) -including the area postrema (AP), Subfornical organ (SFO) and Median eminence (ME)-, choroid plexus and meninges, are proposed as neuroinflammatory-sensor areas^24,25^. These structures rapidly respond to pro-inflammatory stimuli present in the bloodstream including LPS and cytokines^26,27^.

According to these findings, there is no clear evidence how peripheral LPS can directly stimulate the brain, even though the innate immunity receptor TLR-4 signaling pathway in brain cells can be activated by its presence. We hypothesized that LPS is able to enter the brain via those structures that are in contact with blood components, possibly through a plasma-lipoprotein mediated LPS transport into the brain. Hence, the present work is aimed to demonstrate the presence of key elements of LPS, lipid A and core LPS in the brain by immunodetection in physiological conditions and to determine whether this correlates with the TLR-4 signaling pathway in blood-brain interfaces. Also, to investigate whether the LPS transport mechanism is facilitated by plasma lipoproteins and their receptors in the brain. These suggested mechanisms in pshysiological conditions would be useful in further studies of the pathophysiology of neuropsychopathologies related to sterile or low grade of inflammation.

## Results

### Lipid A and core LPS immunosignals were observed in blood-brain interfaces

**Figure 1:**
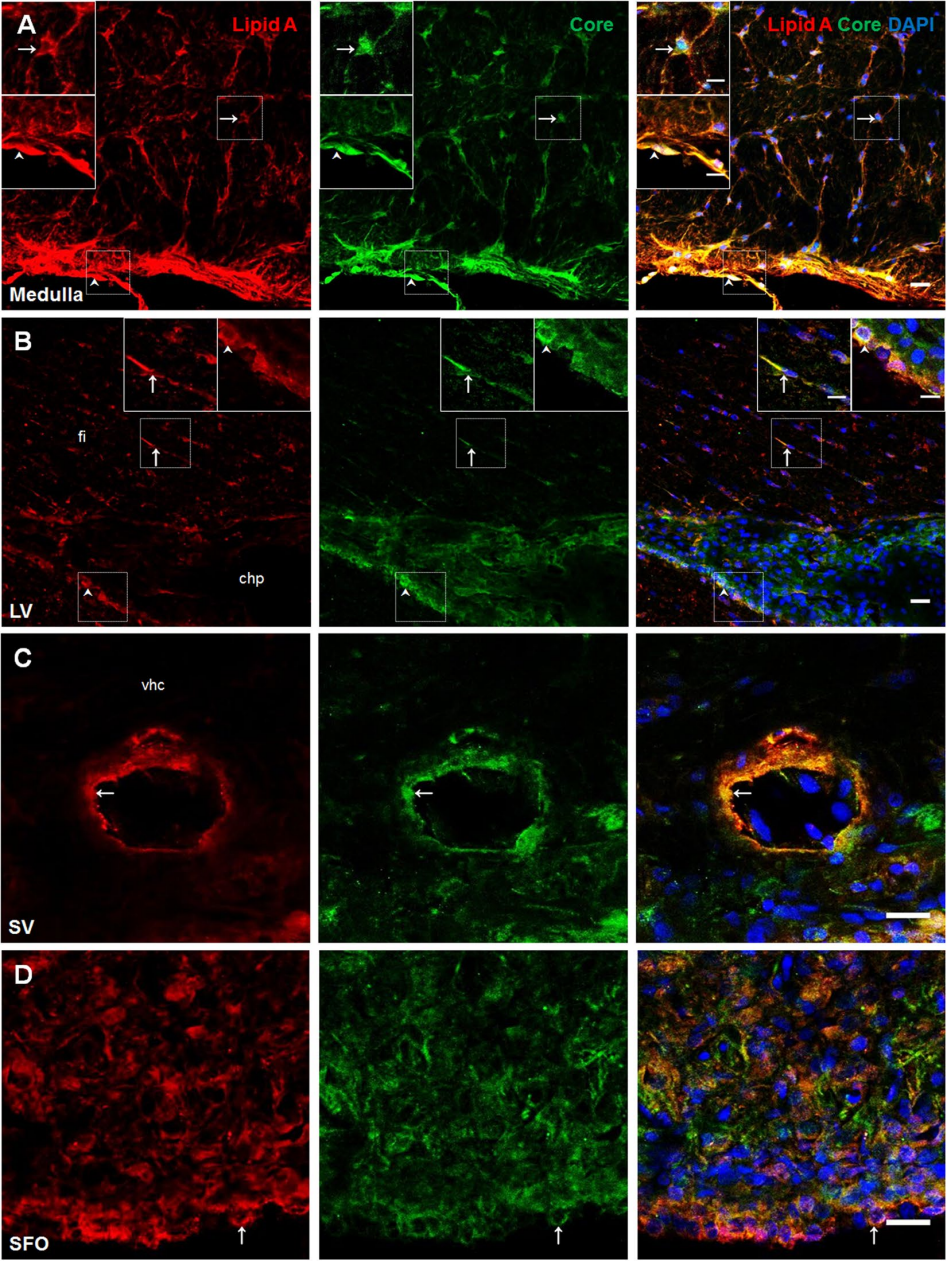
Lipid and Core LPS were present in blood-brain interfaces. Double immunofluorescence of lipid A and Core LPS in rat brain slices were made. (**A**) Medulla oblongata, (**B**) lateral ventricle, (**C**) septal vein and (**D**) subfornical organ. In all cases, red corresponds to lipid A immunosignal, green to Core immunosignal and blue DAPI staining in nucleus. Arrows indicate green and red immunosignal overlapping in astrocyte-like cells (**A,B**), endothelial-like cells (**C**) and ependymal cells and (**D**). Head arrows indicate green and red immunosignals overlapping in meninges (**A**) and tanycyte-like cells (**C**). Choroid plexus (chp), hippocampal fissure (fi), lateral ventricle (LV), subfornical organ (SFO) and septal vein (SV) and ventral hippocampal commissure (vhc). Scale bars = 20 µm, 10 µm for inserts.

To identify the presence of LPS in the brain, two commercial antibodies were used to recognize two regions of LPS with different chemical nature. One polyclonal antibody against lipid A (BP2235, Acris), previously tested in western blot assays by Gibson *et al*.^28^, and a monoclonal antibody against core LPS region (HM6011, Hycult Biotech), previously tested in western blot analysis by Tsuneyoshi *et al*.^29^ and in immunohistochemistry assays by Estes *et al*.^30^. The brain structures analyzed were the medulla oblongata adjacent to meninges, blood vessels (the septal vein), ventricles (lateral and fourth ventricles) and CVOs (subfornical organ, area postrema and median eminence). Our analysis revealed that certain cell types presented immunocolocalization of lipid A and core LPS: (a) In the medulla oblongata, ramified astrocyte-like cells as well as meningeal cells presented a strong immunosignal for both LPS markers (white arrows in Fig. 1A). (b) The septal vein, located in the ventral commissure of the hippocampus, showed co-localization of lipid A and Core markers occurring in endothelial-like cells (Fig. 1B). (c) In the lateral ventricle, structures such as the hippocampal fissure exhibited both immunosignals in astrocyte-like cells in the choroid plexus and tanycyte-like cells surrounding the ventricle wall (Fig. 1C). (d) CVOs also revealed intense immunoreactivity of both LPS markers, in Fig. 1D several cells can be observed in the subfornical organ (SFO), especially ependymal cells in contact with the 3^rd^ ventricle. Supplementary Fig. S1A shows images from the central canal where overlapping of lipid A and Core immunosignals in tanycyte-like cells can be observed. The area postrema and the fenestrated capillaries of the median eminence also showed immunoreactivity to lipid A and Core LPS (see Supplementary Fig. S1B,C).

In order to further confirm the immunosignal of the LPS antibodies against lipid A and core LPS previously mentioned, a third commercial monoclonal antibody against lipid A (ab8467, Abcam). This antibody has been validated by immunocytochemical and western blot techniques in different experimental settings^31,32^. Supplementary Fig. S2 shows lipid A immunosignal of this antibody in ramified astrocyte-like cells and meningeal cells in medulla oblongata. Similar patterns of immunoreactivity were observed in the selected brain structures studied with the other antibodies (data not shown).

### Lipid A and core LPS co-localized with CD14, TLR-4 and NFκB immunosignals in blood-brain interfaces

**Figure 2:**
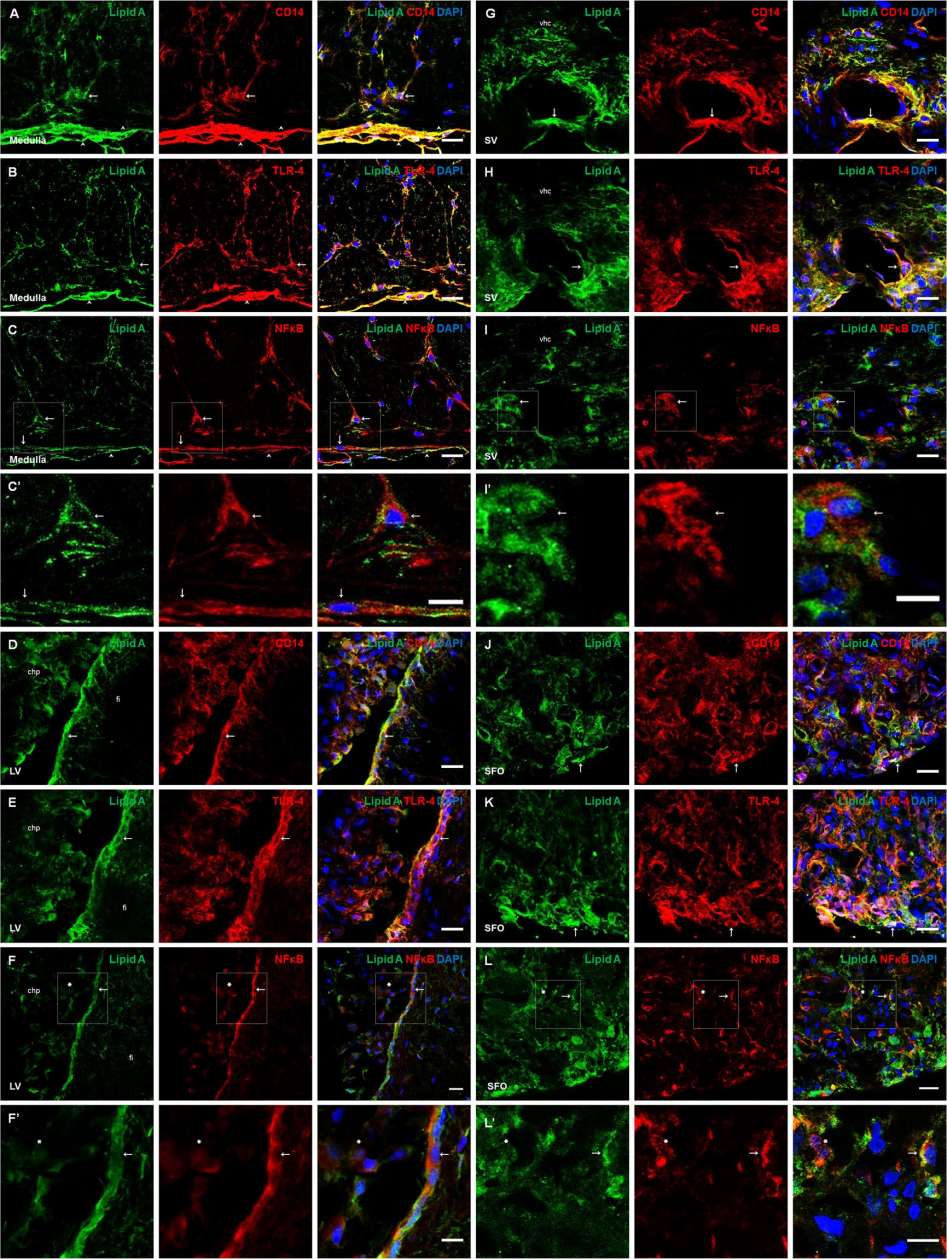
Lipid A was co-localized with the elements of the TLR-4 signaling pathway in blood-brain interfaces. Double immunofluorescence of Lipid A and CD14, TLR-4 or NFκB was made in Medulla oblongata (**A–C**), lateral ventricle (**D–F**), septal vein (**G–I**) and subfornical organ (**J–L**). In all cases green corresponds to Lipid A immunosignal and blue DAPI staining in nucleus. Red immunosignal corresponds to CD14 (**A,D,G,J**), TLR-4 (**B,E,H,K**) and NFκB (**C,F,I,L**, and magnifications C’, F’, I’, L’). Arrows indicate green and red immunosignals overlapping in astrocyte-like cells (**A–C**), tanycyte-like cells from the lateral ventricle (**C–E**), endothelial-like cells (**G–I**) and ependymal cells from the subfornical organ (**J–L**). Head arrows indicate green and red immunosignals overlapping in meninges (**A–C**). Asterisks indicate cells with DAPI and NFκB red immunosignals overlapping (**F**,F’,**L**,L’). Choroid plexus (chp), hippocampal fissure (fi), lateral ventricle (LV), subfornical organ (SFO) and septal vein (SV) and ventral hippocampal commissure (vhc). Scale bars = 20 µm (**A–L**), 10 µm (C’,I’,F’L’).

In order to demonstrate whether lipid A, as the endotoxic region of LPS, colocalize with elements of the basal innate immune response in the brain, the LPS polyclonal antibody (BP2235, Acris) generated against lipid A was used to perform all the following experiments. The immunoreactivity of TLR-4 mediated signaling elements (CD14, TLR-4 and NFκB) was compared with that of the lipid A signal. In the medulla oblongata, lipid A immunoreactivity in association with CD14, TLR-4 and NFκB was observed in ramified astrocyte-like cells and meningeal cells (Fig. 2A–C). In the lateral ventricle, tanycyte-like cells and choroid plexus (Fig. 2D–F), as well as endothelial-like cells from the septal vein (Fig. 2G–I), showed immunoreactivity and co-localization of lipid A with CD14, TLR-4 and NFκB. In the subfornical organ (Fig. 2J–L). Tanycyte-like cells of the central canal and cells of the area postrema and median eminence were positive to the lipid A immunosignal in co-localization with CD14, TLR-4 and NFκB (see Supplementary Fig. S3). Interestingly, in most of the cell types observed, such as astrocyte-like cells, tanycyte-like cells and cells from the CVOs, the NFκB immunosignal was localized in cytoplasm, with no co-localization with the nuclear marker DAPI, suggesting that NFκB nuclear translocation did not occur (Fig. 2C’,F’,I’,L’ and Supplementary Fig. S3C,F,I). However, some cells in the choroid plexus and in CVOs showed NFκB nuclear translocation (Fig. 2F,F’,L,L’).

**Figure 3:**
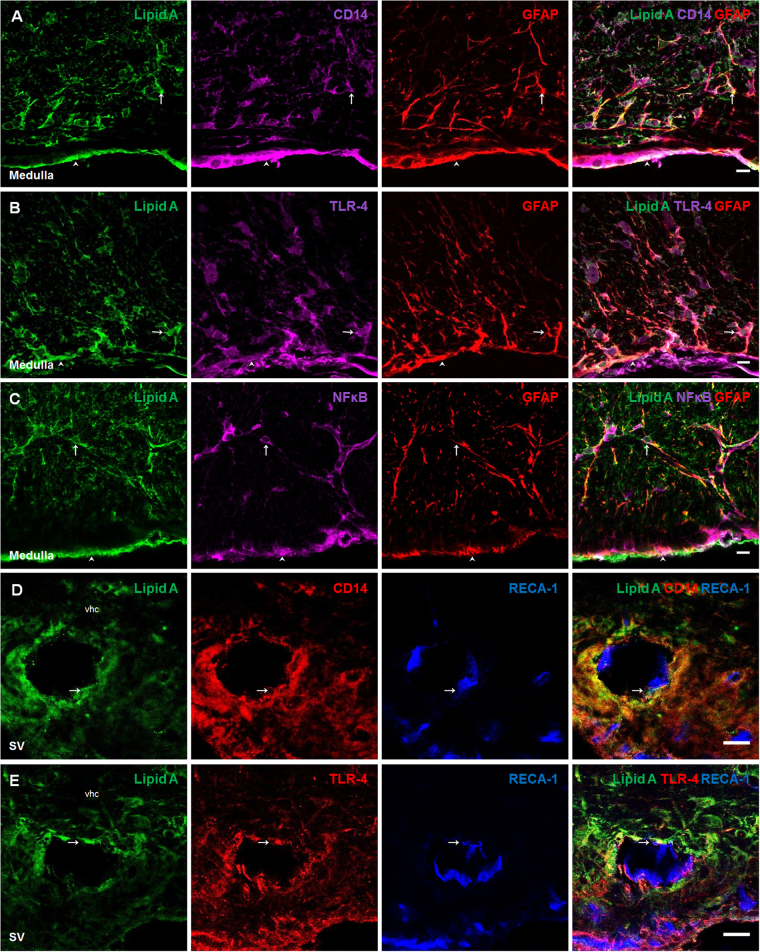
Astrocytes and endothelial cells co-localized Lipid A and the elements of TLR-4 signaling pathway. Triple immunofluoresences of Lipid A, GFAP and CD14, TLR-4 or NFκB; and Lipid A, RECA-1 and CD14 or TLR-4 were made in Medulla oblongata (**A–C**) and septal vein (**D,E**). In all cases, green corresponds to Lipid A immunosignal. Red corresponds to GFAP (**A–C**), CD14 (**D**) and TLR-4 (**E**) respective immunosignals. Magenta corresponds to CD14 (**A**), TLR-4 (**B**) and NFκB (**C**) immunosignals. Blue corresponds to RECA-1 (**D,E**) immunosignal. Arrows indicate green, red and magenta immunosignals overlapping in astrocytes (**A–C**), and green, red and blue immunosignals overlapping in endothelial cells (**D,E**). Head arrows indicate green, red and magenta immunosignals overlapping in meninges (**A–C**). Septal vein (SV). Ventral hippocampal commissure (vhc). Scale bars =20 µm.

In order to confirm the presence of lipid A, as well as the expression of CD14, TLR-4 and NFκB in astrocytes of the medulla oblongata, triple immunofluorescences were performed using GFAP, as astrocytic marker (Fig. 3A–C). In the case of the presence of lipid A in co-localization with CD14 and TLR-4, in endothelial cells, triple immunofluorescences were performed with the endothelial cell marker RECA-1, (Fig. 3D and E, respectively). A similar immunostaining pattern was observed between lipid A, TLR-4 markers and GFAP; the same results were obtained in endothelial cells marked with RECA-1, confirming that astrocytes and endothelial cells have the ability to bind LPS.

The same experiments were carried out in order to study the co-localization of the core LPS and TLR-4 signaling pathway molecules. The immunoreactivity patterns observed were similar to lipid A in all brain structures and cellular types tested (see Supplementary Fig. S4).

### Lipoproteins as a possible transport mechanism for LPS to reach the brain parenchyma

**Figure 4:**
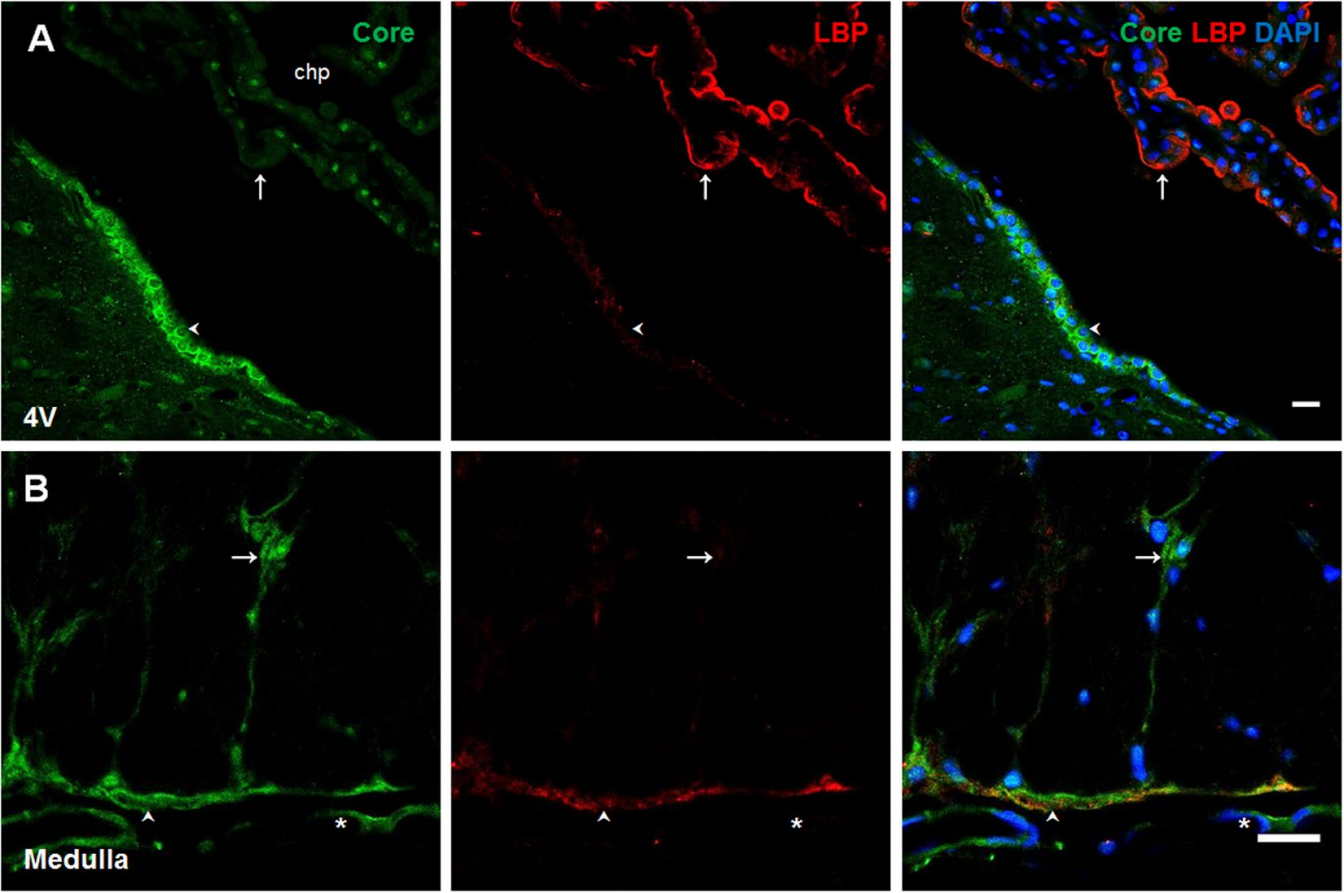
LBP presence in choroid plexus. Double immunofluorescence of core LPS and LBP in the fourth ventricle (**A**) and medulla oblongata (**B**). In all cases green corresponds to core immunosignal, red to LBP and blue DAPI staining in nucleus. Arrows indicate green and red immunosignals overlapping in, choroid plexus (**A**), and in astrocyte-like cells of the medulla oblongata (**B**). Head arrows indicate green and red immunosignals overlapping in tanycyte-like cells from the fourth ventricle (**A**) and in meninges of the medulla oblongata (**B**). Fourth ventricle (4 V). Scale bars = 20 µm.

**Figure 5:**
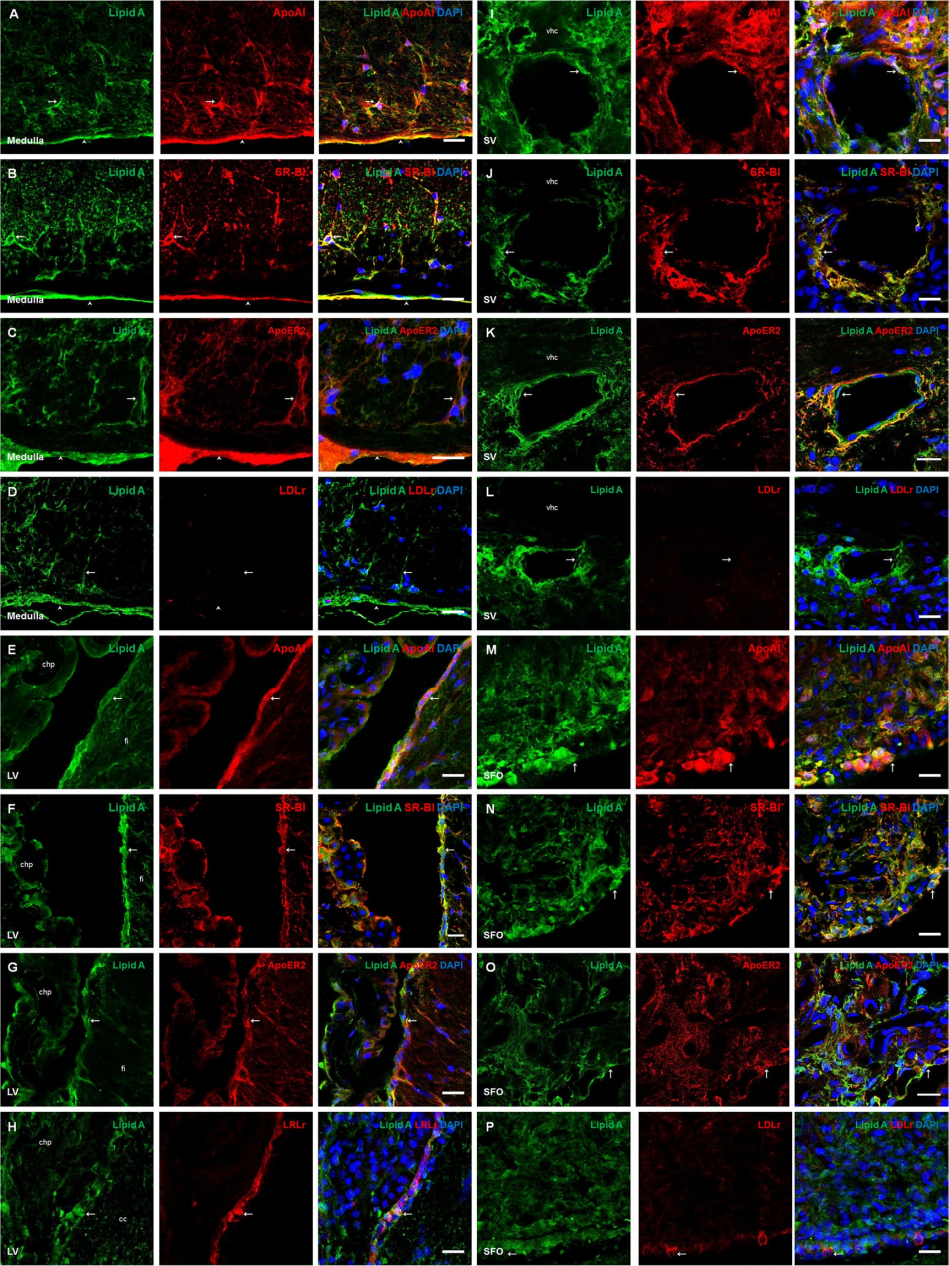
ApoAI and lipoprotein receptors were co-localized with lipid A in blood-brain interfaces. Double immunofluorescence of Lipid A and ApoAI, SR-BI, ApoER2 or LDLr were made in Medulla oblongata (**A–D**), lateral ventricle (**E–H**), septal vein (**I–L**) and subfornical organ (**M–P**). In all cases green corresponds to Lipid A immunosignal and blue DAPI staining in nucleus. Red immunosignal corresponds to ApoAI (**A, E, I** and **M**), SR-BI (**B,F,J** and **N**), ApoER2 (**C,G,K**, and **O**) and LDLr (**D,H,L** and **P**). Arrows indicate green and red immunosignals overlapping in astrocyte-like cells (**A–D**), tanycyte-like cells from the lateral ventricle (**E–H**), endothelial-like cells (**I–L**) and ependymal cells from the subfornical organ (**M–P**). Head arrows indicate green and red immunosignals overlapping in meninges. Corpus callosum (cc), choroid plexus (chp), hippocampal fissure (fi), ventral hippocampal commissure (vhc), subfornical organ (SFO) and septal vein (sv). Scale bars = 20 µm.

After detecting lipid A and core LPS immunosignals in co-localization with TLR-4 pathway elements in the CNS, we further investigate how LPS molecules enter the brain parenchyma. First, an immunofluorescence was performed to identify the pattern of LBP distribution in all the brain areas and cellular types tested. This molecule was only seen in the choroid plexus and with a very slight signal in astrocyte-like cells and meningeal cells in medulla oblongata (Fig. 4). Since LPS has also affinity to apolipoproteins, especially those present in HDL and LDL particles^13^, the distribution of the lipid A immunosignal in relation to the presence of ApoAI, its receptor SR-BI, apolipoprotein E receptor 2 (ApoER2) and low density lipoprotein receptor (LDLr) in the blood-brain interfaces were studied. Interestingly, in the medulla oblongata, the immunosignal of lipid A in co-localization with ApoAI, SR-BI and ApoER2 was identified in astrocyte-like cells and meningeal cells (Fig. 5A–C). LDLr did not show immunoreactivity in these cell types (Fig. 5D). Tanycyte-like cells and the choroid plexus from the lateral ventricle were positive for ApoAI, SR-BI and ApoER2 in co-localization with lipid A (Fig. 5E–G), however, LDLr was present only in tanycyte-like cells in this area (Fig. 5H). In endothelial-like cells from the septal vein, the presence of ApoAI, SR-BI and ApoER2 were observed in co-localization with lipid A (Fig. 5I–K), but again LDLr was not present in this structure (Fig. 5L). In the subfornical organ, several cells showed immunoreactivity to ApoAI, SR-BI and ApoER2, particularly, in ependymal cells in co-localization with lipid A (Fig. 5M–O), and only in some cases of this cell type had immunoreactivity to LDLr (Fig. 5P).

In the central canal, tanycyte-like cells showed an intense immunosignal for all the lipoprotein markers used (see Supplementary Fig. S5A–D). However, in the area postrema and median eminence only ApoAI and SR-BI were observed (see Supplementary Fig. S5E–H).

**Figure 6:**
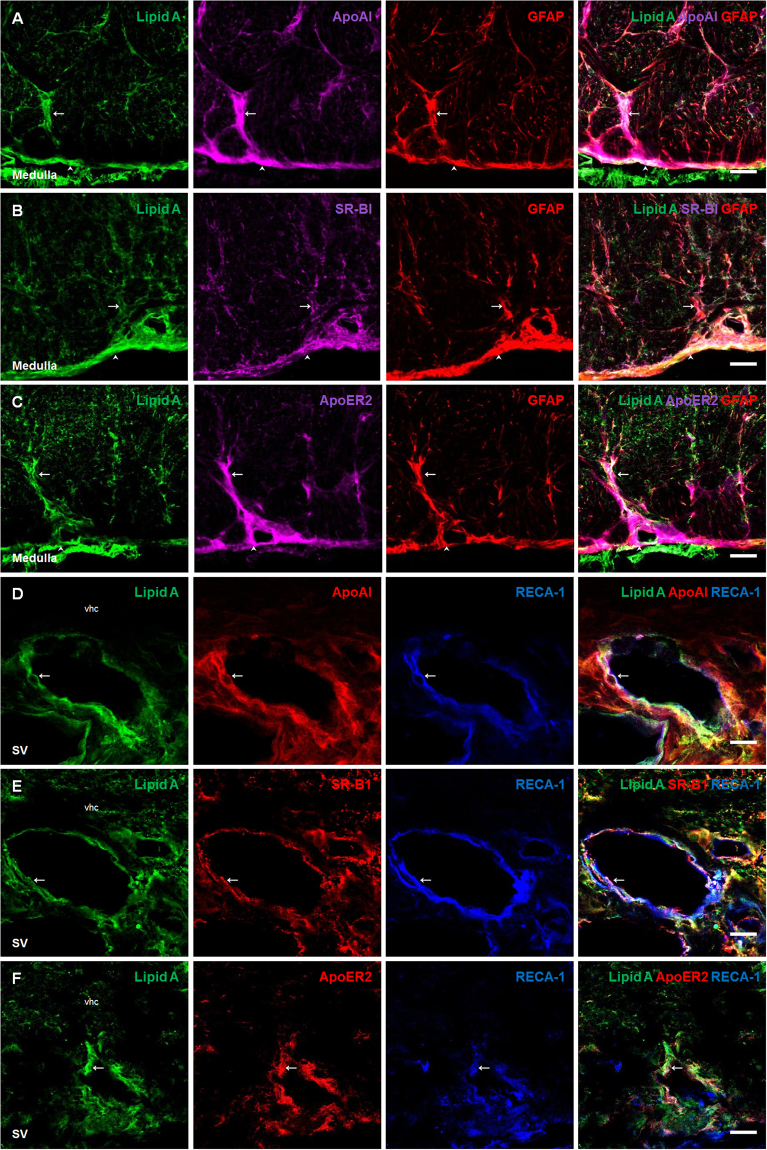
Astrocytes and endothelial cells co-localized Lipid A with ApoAI and lipoprotein receptors. Triple immunofluoresences of Lipid A, GFAP and ApoAI, SR-BI or ApoER2; and Lipid A RECA-1 and ApoAI, SR-BI or ApoER2 were made in Medulla oblongata (**A–C**) and septal vein (**D–F**). In all cases, green corresponds to Lipid A. Red corresponds to GFAP (**A–C**), ApoAI (**D**), SR-BI (**E**) and ApoER2 (**F**) immunosignals. Magenta corresponds to ApoAI (**A**), SR-BI (**B**) and ApoER2 (**C**) immunosignals. Blue corresponds to RECA-1 (**D–F**) immunosignal. Arrows indicate green, red and magenta immunosignals overlapping in astrocytes (**A–C**), and green, red and blue immunosignals overlapping in endothelial cells (**D–F**). Head arrows indicate green, red and magenta immunosignals overlapping in meninges (**A–C**). Septal vein (SV). Ventral hippocampal commissure (vhc). Scale bars = 20 µm.

In order to confirm the presence of lipid A and ApoAI, as well as the expression of SR-BI and ApoER2 molecules in astrocytes of the medulla oblongata, triple immunofluorescences were performed using GFAP (Fig. 6A–C). In the case of the presence of lipid A in co-localization with ApoAI, SR-BI and ApoER2 in endothelial cells, triple immunofluorescences with RECA-1 were performed (Fig. 6D–F). A similar immunostaining pattern was observed for lipid A, lipoprotein markers and GFAP; the same results were obtained in endothelial cells marked with RECA-1, confirming that astrocytes and endothelial cells can bind LPS through lipoprotein transport.

A parallel series of experiments were carried out to check the co-localization of core LPS and ApoAI, SR-BI, ApoER2 and LDLr and similar patterns of immunoreactivity as with lipid A were found in all brain structures and cellular types tested (see Supplementary Fig. S6A–D).

### LPS antibodies tested in different tissues homogenates by Western blot analysis

To confirm the presence of LPS in brain tissue, as well as demonstrated in immunofluoresce assays, a western blot analysis was performed. Purified *E. coli* LPS used as a positive control in contrast from medulla oblongata and frontal cortex homogenates. Also, LPS positive tissues homogenates were used, such as liver due to its function of LPS clearance and excretion through bile^33^, and distal colon because of the presence of LPS as a consequence of microbiota contribution^34^. Homogenate samples from distal colon are part from a study recently published of our research group^35^. These samples were obtained from Wistar rats subjected to a chronic mild stress (CMS) protocol with or without intestinal antibiotic decontamination (2 mg/mL of streptomycin sulfate and 1500 U/mL of penicillin G), and control conditions. Samples derived from animal with intestinal antibiotic decontamination were used as negative control of the presence of LPS in a tissue.

**Figure 7:**
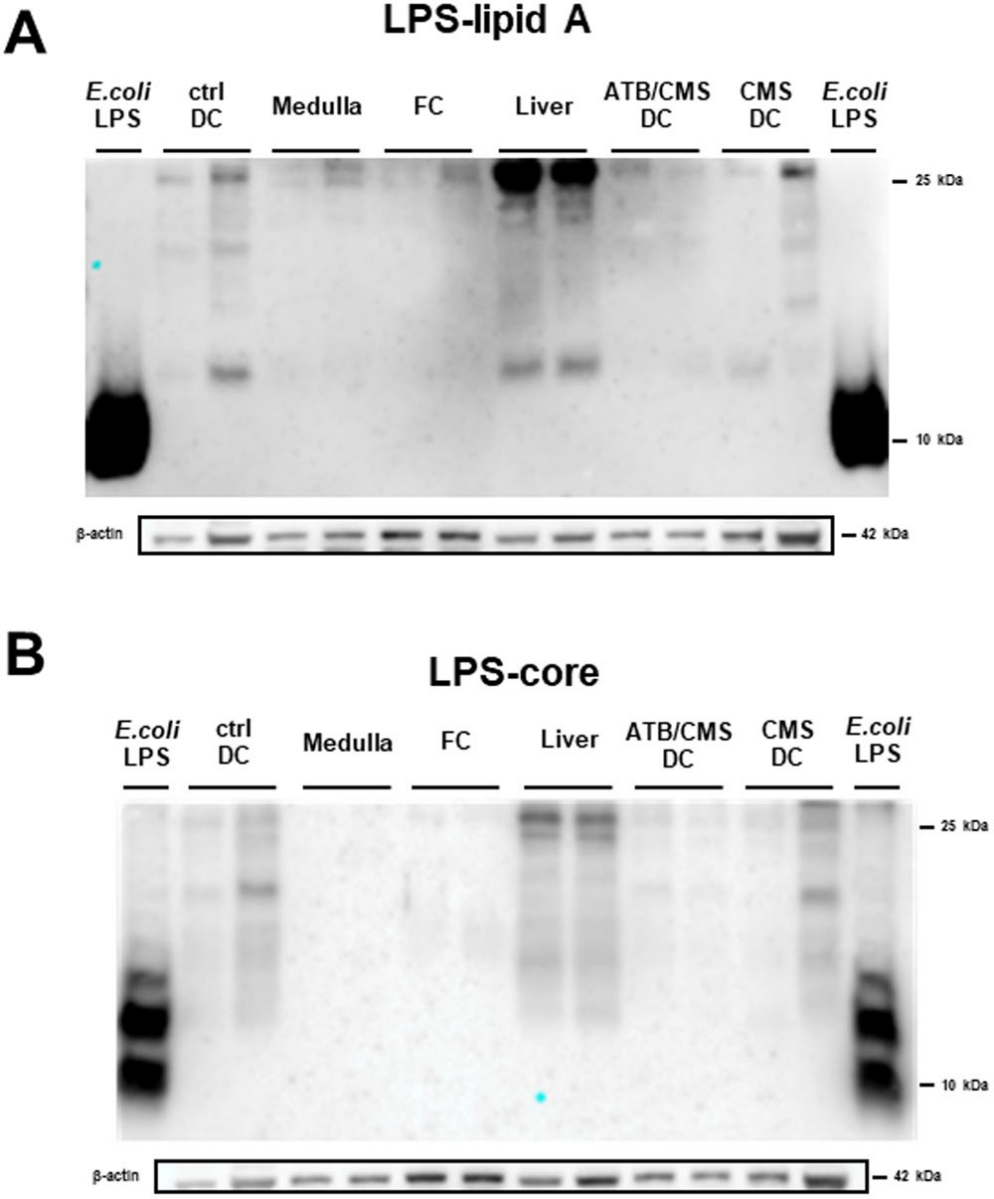
Specific antibodies against lipid A and Core LPS regions can detect the LPS in brain tissue homogenates. (**A**) Western blot of lipid A LPS region in medulla oblongata, frontal cortex (FC), liver and distal colon (DC). (**B**) Western blot of Core LPS region in medulla oblongata, frontal cortex (FC), liver and distal colon (DC). Purified *E. coli* LPS (*E. coli* LPS), Control distal colon (ctrl DC), medulla oblongata (medulla), frontal cortex (PFC), Antibiotic decontamination treatment and chronic mild stress condition distal colon (ATB/ CMS DC), chronic mild stress condition distal colon (CMS DC).

As shown in Fig. 7, lipid A and core LPS antibodies were able to recognize purified *E. coli* LPS, samples of distal colon from rats with an antibiotic decontamination treatment showed reduced immunosignal in comparison with control and from CMS condition rats, liver samples showed the most intense immunosignal. Medulla oblongata and frontal cortex samples were positive for LPS immunodetection but with less intensity compared with positive control tissues. Blots of different molecular weight (MW) were observed in all the tissue samples tested compared to purified *E. coli* LPS that showed a single blot of low MW. Possibly, multiple blots in homogenate tissues are consequence of LPS binding to different MW proteins, it will be discussed further.

### Comparative analysis of MW between LPS and TLR-4, SR-B1, CD14 or ApoAI immunoblots in tissue homogenates

**Figure 8:**
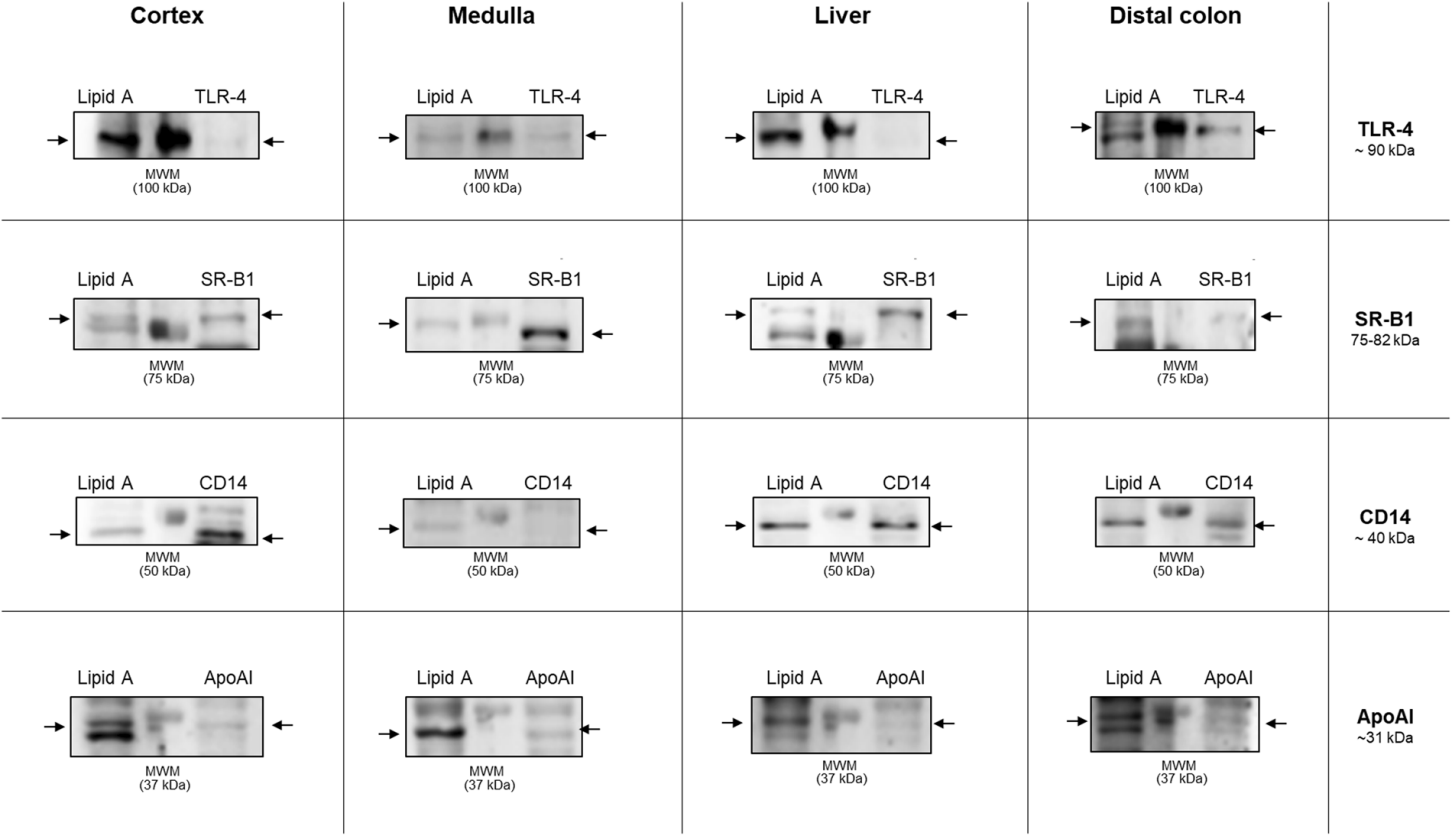
Multiple blots in LPS detection in tissue homogenates by western blot are LPS bound to proteins. Simultaneous western blot analysis for lipid A and CD14, TLR-4 ApoAI or SR-B1 detection in cortex, medulla, liver and distal colon tissue homogenates from rats under control conditions. MWM: molecular weight marker. Arrows indicate blots of similar MW for lipid A and target protein (CD14, TLR-4 ApoAI or SR-B1). Each cropped blot image corresponds to different simultaneous Western blot analysis, individually made for each lipid A and target protein comparison (See original immunoblots in Supplementary Fig. S7).

In order to identify whether these blots correspond to LPS bound to proteins with high affinity to endotoxin, we carried out an experiment consisting in a simultaneous WB analysis to compare the MW of proteins of interest with putative binding capacity to LPS with the blots revealed by lipid A antibody in samples from brain cortex, medulla oblongata, liver and distal colon (see Methods section for details). As shown in Fig. 8, the molecules with affinity for LPS, such as TLR-4 (~90 kDa), SR-B1 (73–82 kDa), CD14 (~40 kDa) and ApoAI (~31 kDa), have the same MW that some of the blots present in LPS immunoreactivity in the different tissue homogenates. In Supplementary Fig. S7 are shown original images of the immunoblotting analysis.

### LPS levels in basal conditions

As a previous question, and in order to clearly establish basal conditions of all the animals used, LPS levels in drinking water, food and plasma were measured by LAL test. LPS levels in drink water were 1.245 (±0.09946) EU/mL, food 443.8 (±119.7) EU/mL and plasma were 0.2528 (±0.02652) EU/mL.

## Discussion

The present study describes the co-localization of lipid A and core LPS with key elements of the TLR-4 signaling pathway and their distribution in the main blood-brain interface regions in physiological conditions. Lipoproteins and their receptors are also present in brain areas and cellular types displaying immunoreactivity for both LPS regions, suggesting a putative mechanism of transport of LPS from the periphery to brain parenchyma. In particular, LPS was found mainly in astrocytes adjacent to meninges and meningeal cells in the medulla oblongata, tanycyte-like cells along the ventricular system, choroid plexus, endothelial cells and CVOs, and always appeared in co-localization with CD14, TLR-4 and NFκB.

In our study, we demonstrated the presence of basal levels of LPS in the plasma by LAL assay, as well as detectable levels of LPS in drinking water and food provided to the rats. In agreement with this, measurable plasma LPS levels of control animals have been detected in other studies^7,35^, as well as in healthy human plasma and tissues^8,9^. Furthermore, the constitutive expression of CD14 and TLR-4 in the CNS has been reported^36^ without any direct evidence of LPS presence in brain as a regulator of their expression and activity. In this way, several attempts to study how peripheral LPS enters the brain used fluorescent^21^ or radioactively labeled LPS molecules^22^ but none of them were able to clearly prove its presence in brain tissue. Some of these studies state that LPS does not cross the BBB and that the neuroinflammation caused by peripheral LPS is mediated by secondary inflammatory molecules, such as nitric oxide (NO) and interleukin 1 (IL-1) released by endothelial cells in brain vessels^23^. However, all of these studies searched for the whole LPS molecule inside the brain instead of the lipid A, which is the main stimulator of the innate immune system through TLR-4^1^. The present series of results indicates that at least lipid A and core LPS can reach the brain, after confirming with three specific antibodies against these two LPS regions, and all of them previously used in different studies with western blot and immunofluorescence techniques as mentioned above in results section.

Both lipid A and core LPS bind to the CD14/TLR-4 complex in different cell types in structures with the presence of BBB, such as astrocytes in the medulla oblongata, tanycytes in ventricular walls, and endothelial cells in the ventral hippocampal commissure. All these cell types form the blood-brain interface: (a) astrocytes as part of immunologic functional barriers at interfaces between non-neural tissue and CNS parenchyma^37^. (b) tanycytes are considered specialized astrocytes^38^ in direct contact with the cerebrospinal fluid (CSF) along the ventricular walls and CVOs regulating the exchange between the blood, brain and CSF^39^. Finally, (c) endothelial cells in brain vessels arbitrate a neuroimmune communication by actively responding to immune challenges such as LPS through the release of cytokines and other mechanisms^40^.

Areas lacking the BBB, such as CVOs, can immediately develop a pro-inflammatory response against LPS since they are in direct contact with blood^25,41^. Similarly, the choroid plexus and meninges have been described as having basal expression of TLR-4 that can be activated by LPS administered intraperitoneally^42^ and lipid A administered intracerebroventricularly^43^. For a long time, it has been investigated and discussed how CVOs respond against a peripheral stimulus^24^. However, this work demonstrates for the first time, to our knowledge, the presence of LPS in these structures. The observation of lipid A and Core LPS immunosignals in tanycyte-like cells from the lateral ventricles and central canal, choroid plexus and CVOs demonstrate that LPS is present along the ventricular system.

Interestingly, in LPS-positive cells, most of the NFκB immunosignal was detected in cytoplasm. Having designed the experiment in naturalistic conditions, without manipulation of animals in the animal facility, it can be assumed that the control, physiological levels of LPS might bind to the constitutive, sentinel innate immune receptor TLR-4 in the CNS^36^ without activating a real inflammatory intracellular pathway. Taking into account that lipid A from different bacteria has different endotoxic properties, depending on their effects as weak TLR-4 agonists and/or antagonists^2,3,8,44^, the lack of NFκB activation in control conditions might reflect a possible mechanism for protecting against toxicity from opportunistic bacteria that deserve further studies.

After having described the presence of particular regions of LPS molecule in different brain structures, a big issue is how LPS is transported to the brain. The best-characterized mechanism is the binding protein LBP. In our naturalistic experimental conditions, LBP immunofluorescence was found in the choroid plexus and with a very slightly signal in astrocytes. Taking into account other proposed alternative transport mechanisms for LPS such as plasma lipoproteins^45^, we have analyzed their possible role as transporters for reaching tanycytes, astrocytes and endothelial cells in the experimental conditions used here, carrying out several immunofluorescence experiments to detect ApoAI, which is the main Apo in plasma HDL particles, its receptor SR-BI, and two of the main lipoprotein receptors in the brain, ApoER2 and LDLr. The results presented here confirm that there is a possible lipid A transport mechanism in the brain mediated by plasma lipoproteins.

The binding of lipoproteins to LPS has been explained as a beneficial homeostatic mechanism because it contributes to neutralizing and clearing LPS toxicity by eliminating LPS through the liver and bile^15^. A pathophysiological role has also been proposed, since a high-fat diet can promote LPS translocation by chylomicron binding, contributing to chronic diet-induced inflammation^46^. On the other hand, the presence of LPS in plasma induces hyperlipidemia by increasing the synthesis of VLDL and promoting pro-inflammatory cytokine production in different tissues^47^. However, HDL is one of the plasma lipoproteins with high affinity for LPS and has anti-inflammatory properties^13,47,48^. By experiments of binding and affinity among HDL particles and different lipid A moiety molecules, it was demonstrated that the phosphates and the diglucosamine backbone functional groups from lipid A interact with HDL^49^. The main apolipoprotein present in HDL particles is ApoAI and is considered the major contributor to HDL-anti-endotoxin function by the ability to bind lipid A in the amphipathic alpha-helix domains, neutralizing LPS toxicity^50,51^. It has been reported that ApoAI transports LPS into hepatocytes for its metabolic degradation, reducing the interaction of LPS with phagocytic cells and, in consequence, preventing pro-inflammatory cytokine synthesis^52^. Another apolipoprotein characterized by its ability to bind LPS is ApoE, present in almost all the lipoproteins, from chylomicrons to LDL, but in rodents is mainly associated with HDL^53,54^. The increase of ApoE in plasma has been reported during infections and sepsis, in animal models and patients, related with a LPS-clearance effect^14,55^. In the case of LDL, its ability to bind LPS by ApoB100 has been also demonstrated^56^.

The role of lipoproteins in the brain differs from the rest of the body; the brain has the ability to produce its own nascent lipoproteins, mainly produced by glial cells, and only small particles of plasma HDL have the ability to cross the BBB^57^. There is also evidence confirming the entry of LDL by transcytosis through the BBB^58^. Some authors affirm that the only plasma HDL-contained apolipoprotein able to cross the BBB is ApoAI, while ApoE and ApoB cannot. The main lipoprotein receptor for ApoAI is SR-BI^59^, involved in the regulation of the inflammatory response against LPS in macrophages^60^. In the brain, HDL particles are up-taken through SR-BI, which is expressed in endothelial^61^ and glial cells^62^. Plasma HDL particles containing ApoAI can lead the formation of new nascent HDL-like lipoproteins by acquiring ApoE, synthesized by glial cells^62,63^. As occurs in plasma, remodeling and maturation in the brain of these new lipoproteins derived from plasma HDL need the participation of several proteins such as cholesteryl ester transfer protein, lecithin:cholesterol acyltransferase and phospholipid transfer protein (PLTP)^63,64^. The latter is another protein with binding ability to LPS molecules that belongs to the LPS binding protein family. In plasma, a PLTP has been described that promotes disaggregation of LPS and transfers it to HDL and between HDL and LDL^64–66^. Mature HDL-like particles can bind to neurons or astrocytes through the binding of ApoE to LDLR family receptors, including ApoER2^62,63^, one of the major lipoprotein receptors expressed in the brain^67^. Astrocytes and neurons are able to express ApoER2 on their membrane surface^57^, especially astrocytes from the medulla^68^.

Taking all these evidences together, we hypothesized three possible mechanisms for a lipoprotein-mediated transport of LPS into the brain across the BBB. First: plasma ApoAI-HDL-bound LPS can cross the BBB via SR-BI-mediated uptake in endothelial cells, tanycytes and/or meninges. Second: ApoAI-HDL-bound LPS can become part of a new nascent lipoprotein with ApoE in astrocytes and then the ApoE of this mature lipoprotein binds to ApoER2. Third: LPS from plasma ApoAI-HDL might be transferred to ApoE by proteins like PLTP, and then mature ApoE-lipoprotein binds to ApoER2. In relation to plasma ApoE-lipoprotein-bound LPS, the possibility of the ApoER2 uptake by endothelial cells cannot be discarded, as we have demonstrated the expression of this receptor in this cell type.

It has been demonstrated that LPS from lipoprotein particles is able to bind to its receptors CD14 and TLR-4, as has been seen to occur in different experimental settings. Lipoproteins have the ability to bind to several receptors at the same time (i.e. CD14/TLR-4 and SR-BI or ApoER2) as occurs in the LPS-LDL complex in macrophages and hepatocytes, where this complex can bind to scavenger receptors, LDLRs and LPS receptors^56,69^. It could be possible that this mechanism can occur also in the SNC. However, it is important to point out the necessity of further studies to elucidate the presence of LPS in the brain using germ free animals and/or knock out animals for the studied molecules of interest.

Finally, another alternative possibility, of special interest in areas lacking the BBB, like CVOs, is that the transport of LPS could be also mediated by supramolecular aggregate structures that can be formed by an amphiphilic molecule like lipid A in an aqueous environment^70,71^, such as in the blood stream^72^. Then LPS can reach the receptors without the necessity of being transported by a binding protein or lipoprotein. However, this possibility needs different chemical/structural approaches.

The immunoblotting analysis carried out to confirm the presence of LPS in brain tissues, demonstrated that in all cases it was identified similar blot pattern among all the tested tissue samples (brain cortex, medulla oblongata, liver, and distal colon) proving that at the same protein concentration (20 µg) liver and distal colon, which are the LPS positive tissues, present higher LPS immunosignal than brain cortex and medulla oblongata tissues. Furthermore, the multiple blot observed in LPS detection by this technic, can be caused by LPS molecules bound to proteins of different MW present in tissue homogenates. This hypothesis can be supported by previous evidence demonstrating that LPS can be bound by a wide range of proteins, mainly, to proteins with cationic profile^73–75^. Unlike, the positive control of *E. coli* LPS, a highly purified molecule dissolved in water, without any other molecule being able to bind, could tend to form supramolecular aggregates in the aqueous environment^70,71^, showing a single blot of low MW compared to tissue homogenates. In consequence, this could reflect that the levels of free LPS in the different tissues tested are very low, and possibly under the detection limits of the Western Blot method here used. Our experiment of simultaneous western blot to compare blots of similar MW between LPS and target proteins with LPS affinity such as CD14, TLR-4, ApoAI and SR-B1 support the possibility that LPS is bound to these proteins in the tissues tested. This event was previously demonstrated in a study by LPS binding assays from cells incubated with LPS, where it was observed by immunoblotting LPS binding activity in several proteins such as TLR-4/MD2 complex and CD14^29^.

In conclusion, the results indicate a role for LPS in physiological circumstances as an intermediate between microbiota and brain. We have found a particular methodology for detecting LPS in the CNS that could be used to further study the innate immunity driven neuroinflammatory response elicited in different pathological conditions in which peripheral LPS levels vary, such as infections and stress-related neuropathologies. In addition, we outlined a lipoprotein-related mechanism of transport of LPS into the CNS although its apparent complexity requires further studies to fully characterize and to investigate its potential pathological or therapeutical role.

## Methods

### Animals

Eleven male Wistar Hannover rats, weighing 250–280 g were used. All experimental protocols adhered to the guidelines of the Animal Welfare Committee of the Universidad Complutense de Madrid in accordance with European Legislation (2010/63/EU) and Dept. Health & Human services, NIH USA, (statement of compliance of standards OLAW: #A5635-01) (The ethics committee of Universidad Complutense de Madrid approved the study, ref. PROEX 421/15, Feb 10th 2016). The rats were housed individually (cage type 1000, 215 × 465 × 145 mm, 1000 cm^2^, Panlab, Spain) with standard temperature and humidity conditions and in a 12-h light/ dark cycle (lights on at 8:00 h) in wood bedding (ECO-PURE chips, Datesand, Manchester, UK, autoclaved) with free access to food (Teklad Global protein rodent diet 2018, Envigo, Spain) and water (tap water, filtered 5 µm). All the animals were maintained under standard conditions seven days prior to the experiment.

### Preparation of biological samples

All animals were euthanized with sodium pentobarbital (320 mg/Kg i.p.) at 9:00–10:00. Blood samples, for plasma determinations, were obtained by cardiac puncture using a syringe loaded with 3.15% (w/v) trisodium citrate solution, and then plasma was separated by centrifugation at 402 g for 10 min at 4 °C. Samples were stored at -80 °C until assayed. Seven animals were employed for transcardial perfusion performed through the left ventricle with 200 mL sterile saline solution, and the right atrium was opened, next 200 mL of 4% paraformaldehyde (PFA) in 0.1 M PBS (pH 7.4). Brains were dissected and post-fixed in 4% PFA overnight at 4 °C, then were equilibrated in 30% sucrose at 4 °C until precipitate, around 48 h. Brains were embedded in optimum cutting temperature (O.C.T. tissue tek) and frozen at −20 °C to obtain 15 μm coronal sections using a cryostat (Leica CM1950). Consecutive sections were obtained from three regions of the brain where CVOs and other blood-brain interfaces are present: the SFO (between Bregma −0.84 and −1.08 mm), the ME (between Bregma −1.80 and −2.04 mm) and AP (between Bregma −13.68 mm to 14.16 mm), using a rat brain stereotaxic atlas as reference^76^. Sections were collected and mounted onto slides with adherent coating. All brain tissue sections were maintained at −40 °C until use. Four animals were used in order to obtain medulla oblongata, frontal cortex, and liver right lobule homogenates. These tissue samples were excised, frozen and stored at −80 °C before assays. Samples were homogenized by sonication in phosphate buffer saline (pH 7) and adding protease inhibitor cocktail (Complete, Roche, Madrid, Spain), homogenates were centrifuged at 12000 g for 10 min, 4 °C. Supernatants were separated and stored at −80 °C before assays.

### Immunofluorescence

Double and triple, simultaneous or sequential immunostainings were performed in at least three consecutive sections for each structure. Sections were washed with KPBS 0.02 M (pH 7.4) for 5 min, treated with 0.1 M glycine during 20 min to eliminate autofluorescence, two washes of 5 min with KPBS, then blocking process for 30 min with 10% BSA, 0.1% triton 100x in KPBS (blocking solution). The incubation with primary antibodies, diluted in blocking solution, was for 2 h at room temperature (all primary antibodies used are listed in Supplementary Table S1). Sections were washed in KPBS three times for 5 min each. The incubation with secondary antibodies, diluted in blocking solution, was for 1 h at room temperature (all secondary antibodies used are listed in Supplementary Table S2). Thereafter, sections were washed three times in KPBS for 5 min each. One last wash was done with deionized water alone, for 5 min. Finally, 4′,6-diamidino-2-phenylindole dihydrochloride (DAPI) containing Fluoroshield^®^ (Sigma Aldrich) mounting medium was added on the slides for the double immunostaing procedure. Fluoroshield^®^ alone was used in the triple immunostaining procedure. Sections were coverslipped and frozen at −20 °C or immediately visualized on a high-performance Nikon Eclipse Ti Series TI-FL Epi-fl Illuminator^®^ epi-fluorescence microscope. Confocal images were obtained in the confocal Olympus microscope FV1200. Images were processed for adjust brightness, contrast and merge images in the processing package “Fiji”. In order to corroborate if unspecific binding of fluorescent secondary antibodies was present, negative controls were carried out, for each antibody, using the sample protocol, instead of primary antibody incubation, tissue was maintained with blocking solution during this time.

### Western blot analysis for LPS antibodies validation

Protein levels were determined using Bradford analysis^77^ of the homogenates from distal colon, liver, medulla oblongata and brain frontal cortex. Homogenates were mixed with Laemmli sample buffer (Bio-Rad, USA) (SDS 10%, distilled H_2_O, 50% glycerol, 1 M Tris HCl, pH 6.8, dithiothreitol and Bromophenol Blue) with β-mercaptoethanol (50 µL/mL Laemmli), 20 µl (1 µg/µL) and LPS purified from *E. coli* (*E. coli* serotype 0111:B4, ref. L2630 from Sigma-Aldrich, Spain) 1 µg, dissolved in endotoxin free water, were loaded too into a 18% SDS-polyacrylamide electrophoresis gel (SDS-PAGE). Once separated on the basis of MW gels were blotted onto nitrocellulose membrane with a trans-blot turbo transfer system (Bio-Rad^®^). Membranes were incubated with specific antibodies, goat polyclonal anti-lipid A IgG (BP2235, Acris) used at a 1:500 dilution and mouse monoclonal anti-core IgG2A (HM6011, Hycult Biotech) used at a 1:500 dilution, overnight at 4 °C. Goat polyclonal anti-lipid A IgG was recognized with a horseradish peroxidase-linked anti-goat IgG, incubated for 60 min at room temperature and revealed by ECL™-kit following manufacturer’s instructions (Amersham Ibérica, Spain). Blots were imaged using an Odyssey^®^ Fc System (Li-COR Biosciences). Membranes incubated with mouse monoclonal anti-core IgG2A were recognized in a sequential secondary antibody incubation for being reveled, first primary antibody was recognized with goat polyclonal anti-mouse IgG2A (A21131, Life Technologies) in an incubation for 60 min at room temperature, and then reveled with horseradish peroxidase-linked anti-goat IgG incubated for 60 min at room temperature. β-actin was used as loading control.

### Simultaneous western blot analysis for corresponding LPS and CD14, TLR-4, ApoAI or SR-B1 blots

Tissue homogenates samples of brain cortex, medulla oblongata, liver and distal colon from non-treated animals, were run on a SDS-PAGE. Loading order was performed as follow: sample 1/MWM/sample 1, in three consecutive lanes. After transfer step, nitrocellulose membrane was cut vertically half the MWM lane, obtaining two membrane sections. Each membrane section was incubated separately, one for lipid A detection and the other one for target protein (CD14, TLR-4, ApoAI or SR-B1). Image acquisition after reveal step was performed placing two membrane sections together, using as reference MWM blots. 18% polyacrylamide gels were used for Lipid A/CD14 and lipid A/ApoAI Western blos. 8% polyacrylamide gels were used for Lipid A/TLR-4 and lipid A/SR-B1 Western blots.

### LPS levels

Drinking water and food pellets from the cages in which the animals used in the study as well as plasma from animals were evaluated to determine LPS levels through *Limulus* amebocyte lysate (LAL) test, using commercially available kits following the manufacturer’s instructions (Hycult Biotech, Uden, The Netherlands). 100 mg of food pellet were suspended in 1 mL of sterile saline solution and incubated at 37 °C for 10 min, in order to soften the sample, then homogenized and finally centrifuged at 3000 rpm for 5 min. Supernatant was recovered for analysis.

## Electronic supplementary material

Supplementary information

## References

[1] Molinaro A (2015). Chemistry of Lipid A: At the Heart ofInnate Immunity. Chem. A Eur. J..

[2] Raetz CRH, Reynolds CM, Trent MS, Bishop RE (2007). Lipid A Modification Systems in Gram-Negative Bacteria. Annu. Rev. Biochem..

[3] Needham BD, Trent MS (2013). Fortifying the barrier: the impact of lipid A remodelling on bacterial pathogenesis. Nat. Rev. Microbiol..

[4] Netea MG, van Deuren M, Kullberg BJ, Cavaillon J-M, Van der Meer JWM (2002). Does the shape of lipid A determine the interaction of LPS with Toll-like receptors?. Trends Immunol..

[5] Moreira AP (2012). de C. Influence of a high-fat diet on gut microbiota, intestinal permeability and metabolic endotoxaemia. Br. J. Nutr..

[6] Roberts, J. D. *et al*. An Exploratory Investigation of Endotoxin Levels in Novice Long Distance Triathletes, and the Effects of a Multi-Strain Probiotic/Prebiotic, Antioxidant Intervention. *Nutrients* **8**, 10.3390/nu8110733 (2016). 10.3390/nu8110733 PMC5133117 27869661

[7] Gárate I (2012). Stress-Induced Neuroinflammation: Role of the Toll-Like Receptor-4 Pathway. Biol. Psychiatry.

[8] Munford RS (2016). Endotoxemia–menace, marker, or mistake?. J. Leukoc. Biol..

[9] Nádházi Z, Takáts A, Offenmüller K, Bertók L (2002). Plasma endotoxin level of healthy donors. Acta Microbiol. Immunol. Hung..

[10] Schumann RR (1990). Structure and function of lipopolysaccharide binding protein. Science.

[11] Selkirk GA, Mclellan TM, Wright HE, Rhind SG (2008). Mild endotoxemia, NF-kappaB translocation, and cytokine increase during exertional heat stress in trained and untrained individuals. Am. J. Physiol. - Regul. Integr. Comp. Physiol..

[12] Waser M (2004). Determinants of endotoxin levels in living environments of farmers’ children and their peers from rural areas. Clin. Exp. Allergy.

[13] Murch O, Collin M, Hinds CJ, Thiemermann C (2007). Lipoproteins in inflammation and sepsis. I. Basic science. Intensive Care Med..

[14] Van Oosten M (2001). Apolipoprotein E protects against bacterial lipopolysaccharide-induced lethality. J. Biol. Chem..

[15] Vreugdenhil ACE, Snoek AMP, van’t Veer C, Greve J-WM, Buurman WA (2001). LPS-binding protein circulates in association with apoB-containing lipoproteins and enhances endotoxin-LDL/VLDL interaction. J. Clin. Invest..

[16] Beck WHJ (2013). Apolipoprotein A-I binding to anionic vesicles and lipopolysaccharides: role for lysine residues in antimicrobial properties. Biochim. Biophys. Acta..

[17] Pirillo A, Catapano AL, Norata DG (2015). HDL in Infectious Diseases and Sepsis. Handb. Exp. Pharmacol..

[18] Maes M, Leunis JC (2008). Normalization of leaky gut in chronic fatigue syndrome (CFS) is accompanied by a clinical improvement: effects of age, duration of illness and the translocation of LPS from gram-negative bacteria. Neuro Endocrinol. Lett..

[19] Maes M, Kubera M, Leunis JC (2008). The gut-brain barrier in major depression: intestinal mucosal dysfunction with an increased translocation of LPS from gram negative enterobacteria (leaky gut) plays a role in the inflammatory pathophysiology of depression. Neuro Endocrinol. Lett..

[20] García Bueno B, Caso JR, Madrigal JLM, Leza JC (2016). Innate immune receptor Toll-like receptor 4 signalling in neuropsychiatric diseases. Neurosci. Biobehav. Rev..

[21] Li Z, Blatteis CM (2004). Fever onset is linked to the appearance of lipopolysaccharide in the liver. J. Endotoxin Res..

[22] Banks WA, Robinson SM (2011). Minimal penetration of lipopolysaccharide across the murine blood-brain barrier. Brain Behav. Immun..

[23] Singh AK, Jiang Y (2004). How does peripheral lipopolysaccharide induce gene expression in the brain of rats?. Toxicology.

[24] Khandaker GM (2015). Inflammation and immunity in schizophrenia: implications for pathophysiology and treatment. Lancet Psychiatry.

[25] Morita S (2016). Heterogeneous vascular permeability and alternative diffusion barrier in sensory circumventricular organs of adult mouse brain. Cell Tissue Res..

[26] Bette M, Kaut O, Schäfer MKH, Weihe E (2003). Constitutive expression of p55TNFR mRNA and mitogen-specific up-regulation of TNF alpha and p75TNFR mRNA in mouse brain. J. Comp. Neurol..

[27] Nadjar A (2003). Nuclear factor kappaB nuclear translocation as a crucial marker of brain response to interleukin-1. A study in rat and interleukin-1 type I deficient mouse. J. Neurochem..

[28] Gibson DL (2006). Salmonella Produces an O-Antigen Capsule Regulated by AgfD and Important for Environmental Persistence. J. Bacteriol..

[29] Tsuneyoshi N (2005). The functional and structural properties of MD-2 required for lipopolysaccharide binding are absent in MD-1. J. Immunol..

[30] Estes, J. D. *et al*. Damaged Intestinal Epithelial Integrity Linked to Microbial Translocation in Pathogenic Simian Immunodeficiency Virus Infections. *PLoS Pathog*. **6**, 10.1371/journal.ppat.1001052 (2010). 10.1371/journal.ppat.1001052 PMC2924359 20808901

[31] Martin I, Cabán-hernández K, Figueroa-santiago O, Espino AM (2015). Fasciola hepatica Fatty Acid Binding Protein Inhibits TLR4 Activation and Suppresses the Inflammatory Cytokines Induced by Lipopolysaccharide *In Vitro* and *In Vivo*. J. Immunol..

[32] Kadowaki T, Inagawa H, Kohchi C, Hirashima M, Soma G-I (2011). Functional Characterization of Lipopolysaccharide derived from Symbiotic Bacteria in Rice as a Macrophage-activating Substance. Anticancer Res..

[33] Topchiy E (2016). Lipopolysaccharide Is Cleared from the Circulation by Hepatocytes via the Low Density Lipoprotein Receptor. PLoS One.

[34] Szabo G, Bala S, Petrasek J (2010). & Gattu, A. Gut-Liver Axis and Sensing Microbes. Dig. Dis..

[35] Martín-Hernández D (2016). Bacterial translocation affects intracellular neuroinflammatory pathways in a depression-like model in rats. Neuropharmacology.

[36] Nguyen MD, Julien J, Rivest S (2002). Innate immunity: the missing link in neuroprotection and neurodegeneration?. Nat. Rev. Neurosci..

[37] Sofroniew MV (2015). Astrocyte barriers to neurotoxic inflammation. Nat. Rev. Neurosci..

[38] Kettenmann, H. & Verkhratsky, A. In *Neuroscience in the 21st Century* 475–506, 10.1007/978-1-4614-1997-6 (Springer New York, 2013).

[39] Langlet F, Mullier A, Bouret SG, Prevot V, Dehouck B (2014). Tanycyte-Like Cells Form a Blood–Cerebrospinal Fluid Barrier in the Circumventricular Organs of the Mouse Brain. J. Comp. Neurol..

[40] Verma S, Nakaoke R, Dohgu S, Banks WA (2006). Release of cytokines by brain endothelial cells: A polarized response to lipopolysaccharide. Brain. Behav. Immun..

[41] Nakano Y (2015). Astrocytic TLR4 expression and LPS-induced nuclear translocation of STAT3 in the sensory circumventricular organs of adult mouse brain. J. Neuroimmunol..

[42] Chakravarty S, Herkenham M (2005). Toll-Like Receptor 4 on Nonhematopoietic Cells Sustains CNS Inflammation during Endotoxemia, Independent of Systemic Cytokines. J. Neurosci..

[43] Takano M (2015). Lipid A-activated inducible nitric oxide synthase expression vianuclear factor-B in mouse choroid plexus cells. Immunol. Lett..

[44] Michaud J (2013). Toll-like receptor 4 stimulation with the detoxified ligand monophosphoryl lipid A improves Alzheimer’s disease-related pathology. Proc. Natl. Acad. Sci. USA.

[45] Wendel M, Paul R, Heller AR (2007). Lipoproteins in inflammation and sepsis. II. Clinical aspects. Intensive Care Med..

[46] Ghoshal S (2009). Chylomicrons promote intestinal absorption of lipopolysaccharides. J. Lipid Res..

[47] Feingold KR (1992). Endotoxin rapidly induces changes in lipid metabolism that produce hypertriglyceridemia: low doses stimulate hepatic triglyceride production while high doses inhibit clearance. J. Lipid Res..

[48] Foit L, Thaxton CS (2016). Synthetic high-density lipoprotein-like nanoparticles potently inhibit cell signaling and production of in fl ammatory mediators induced by lipopolysaccharide binding Toll-like receptor 4. Biomaterials.

[49] Brandenburg K (2002). Biophysical characterization of the interaction of high-density lipoprotein (HDL) with endotoxins. Eur. J. Biochem..

[50] Ma J, Liao X, Lou B, Wu M (2004). Role of Apolipoprotein A-I in Protecting against Endotoxin Toxicity. Acta Biochim. Biophys. Sin. (Shanghai)..

[51] Li Y, Dong J, Wu M (2008). Human ApoA-I overexpression diminishes LPS-induced systemic in fl ammation and multiple organ damage in mice. Eur. J. Pharmacol..

[52] Sumenkova DV, Polyakov LM, Panin LE (2013). Apolipoprotein A-I as a carrier of lipopolysaccharide into rat hepatocytes. Bull. Exp. Biol. Med..

[53] Berbée JFP, Havekes LM, Rensen PCN (2005). Apolipoproteins modulate the inflammatory response to lipopolysaccharide. J. Endotoxin Res..

[54] Li L, Thompson PA, Kitchens RL (2008). Infection induces a positive acute phase apolipoprotein E response from a negative acute phase gene: role of hepatic LDL receptors. J. Lipid Res..

[55] Fu P (2014). Elevated serum ApoE levels are associated with bacterial infections in pediatric patients. J. Microbiol. Immunol. Infect..

[56] Viktorov AV, Yurkiv VA (2005). Binding of LPS and LPS–LDL complexes to rat hepatocytes. Bull. Exp. Biol. Med..

[57] Wang H, Eckel RH (2014). What are lipoproteins doing in the brain?. Trends Endocrinol. Metab..

[58] Dehouck B (1997). A New Function for the LDL Receptor: Transcytosis of LDL across the Blood–Brain Barrier. J. Cell Biol..

[59] Yu C, Youmans KL, Ladu MJ (1801). Proposed mechanism for lipoprotein remodelling in the brain. Biochim. Biophys. Acta.

[60] Cai L, Wang Z, Meyer JM, Ji A, Westhuyzen DR (2012). Van Der. Macrophage SR-BI regulates LPS-induced pro-infl ammatory signaling in mice and isolated macrophages. J. Lipid Res..

[61] Balazs Z (2004). Uptake and transport of high-density lipoprotein (HDL) and HDL-associated a -tocopherol by an *in vitro* blood – brain barrier model. J. Neurochem..

[62] Hottman DA, Chernick D, Cheng S, Wang Z, Li L (2014). HDL and cognition in neurodegenerative disorders. Neurobiol. Dis..

[63] Vitali C, Wellington CL, Calabresi L (2014). HDL and cholesterol handling in the brain. Cardiovasc. Res..

[64] Azzam KM, Fessler MB (2013). Crosstalk Between Reverse Cholesterol Transport and Innate Immunity. Trends Endocrinol. Metab..

[65] Levels JHM (2005). Lipopolysaccharide Is Transferred from High-Density to Low-Density Lipoproteins by Lipopolysaccharide-Binding Protein and Phospholipid Transfer Protein. Infect. Immun..

[66] Gautier T, Lagrost L (2011). Plasma PLTP (phospholipid-transfer protein): an emerging role in “ reverse lipopolysaccharide transport” and innate immunity. Biochem. Soc. Trans..

[67] Reddy SS, Connor TE, Weeber EJ, Rebeck W (2011). Similarities and differences in structure, expression, and functions of VLDLR and ApoER2. Mol. Neurodegener..

[68] Pitas RE, Boyles JK, Lee SH, Hui D, Weisgraber KH (1987). Lipoproteins and Their Receptors in the Central Nervous System. J. Biol. Chem..

[69] Victorov AV (1989). Composition and structure of lipopolysaccharide-human plasma low density lipoprotein complex. Analytical ultracentrifugation, 31P-NMR, ESR and fluorescence spectroscopy studies. Biochim. Biophys. Acta.

[70] Mueller M (2004). Aggregates are the biologically active units of endotoxin. J. Biol. Chem..

[71] Luo Y, Wu ZW, Tsai H, Lin S, Lin P (2015). Endotoxin Nanovesicles: Hydrophilic Gold Nanodots Control Supramolecular Lipopolysaccharide Assembly for Modulating Immunological Responses. Nano Lett..

[72] Santos NC, Silva AC, Castanho MARB, Martins-Silva J, Saldanha C (2003). Evaluation of lipopolysaccharide aggregation by light scattering spectroscopy. ChemBioChem.

[73] Petsch D, Deckwer W, Anspach FB (1998). Proteinase K Digestion of Proteins Improves Detection of Bacterial Endotoxins by the Limulus Amebocyte Lysate Assay: Application for Endotoxin Removal from Cationic Proteins. Anal. Biochem..

[74] Brandenburg K (2003). Cross-linked Hemoglobin Converts Endotoxically Inactive Pentaacyl Endotoxins into a Physiologically Active Conformation. J. Biol. Chem..

[75] Jürgens G (2002). Investigation into the interaction of recombinant human serum albumin with Re-lipopolysaccharide and lipid A. J. Endo.

[76] Paxinos, G. & Watson, C. *The rat brain in stereotaxic coordinates*. (Burlington: Elsevier Academic Press, 2005).

[77] Bradford MM (1976). A Rapid and Sensitive Method for the Quantitation Microgram Quantities of Protein Utilizing the Principle of Protein-Dye Binding. Anal. Biochem..

