# Supplementary material for: Lipopolysaccharide enters the rat brain by a lipoprotein-mediated transport mechanism in physiological conditions
Source: Sci Rep. 2017 Oct 13;7:13113. doi: 10.1038/s41598-017-13302-6 (PMC5640642; doi:10.1038/s41598-017-13302-6)
Supplement: Supplementary file 1 — Supplementary information [file 41598_2017_13302_MOESM1_ESM.pdf]

Lipopolysaccharide enters the rat brain by a lipoprotein-mediated transport mechanism in physiological conditions

Alejandra Vargas-Caraveo[a,b,*], Aline Sayd[a,c], Sandra R Maus[a,c], Javier R Caso[a,c], José LM Madrigal[a,c], Borja García-Bueno[a,c] & Juan C. Leza[a,c]

[a] Dept. of Pharmacology, Faculty of Medicine, Hospital 12 de Octubre Imas12, IUINQ, University Complutense, Madrid, 28040, Spain

[b] Escuela Nacional de Medicina y Homeopatía, Instituto Politécnico Nacional, Mexico city, 07320, Mexico

[c] Centro de Investigación Biomédica en Red de Salud Mental, CIBERSAM. Spain.

* Corresponding author: alejvarg@ucm.es

+34 91394 1478 / 63

SUPPLEMENTARY INFORMATION

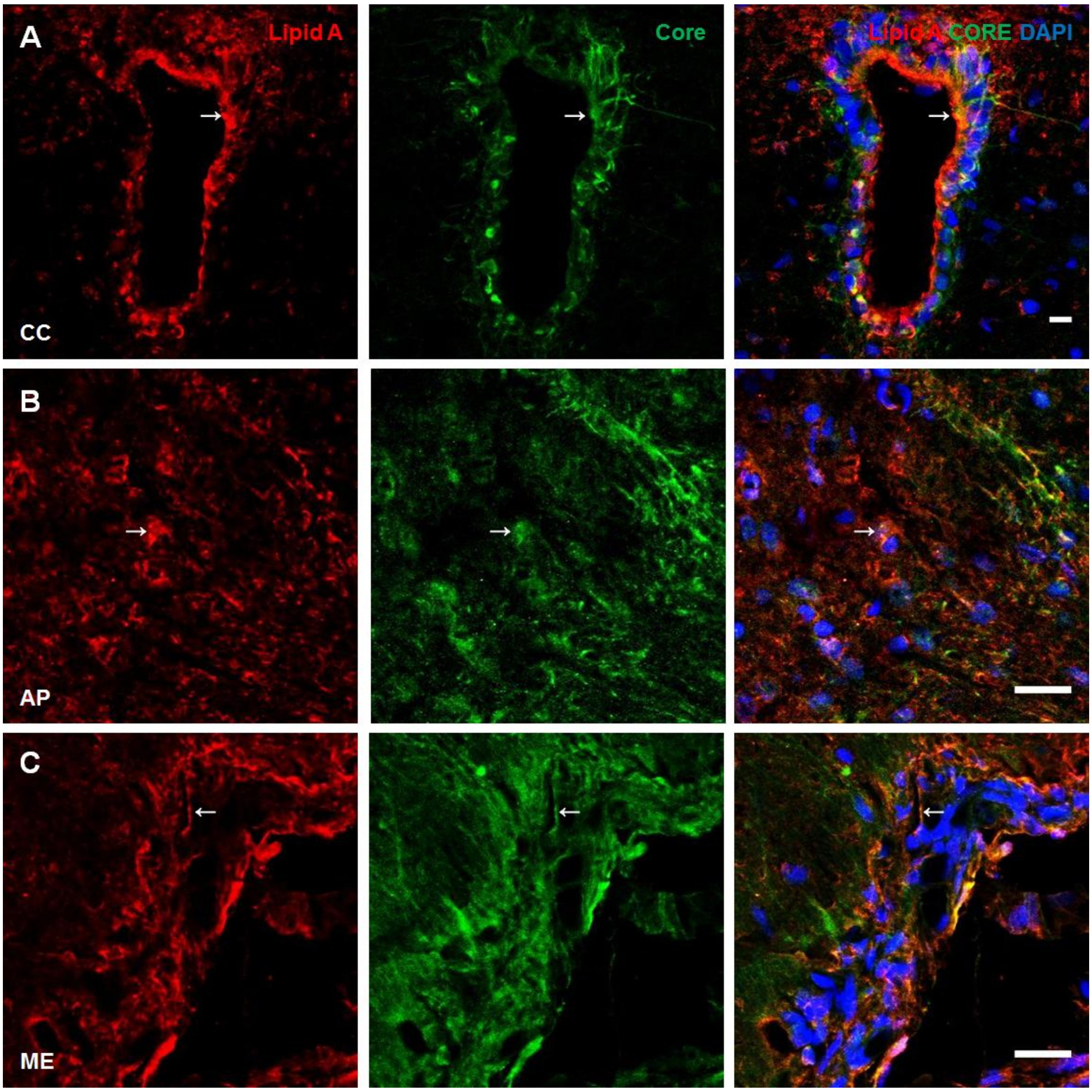

Figure S1. Lipid A and Core LPS were present in tanycyte-like cells from the central canal and circumventricular organs. Double immunofluorescence of lipid A and Core LPS in the central canal (A), area postrema (B) and median eminence (C). In all cases, red corresponds to lipid A immunosignal, green to Core immunosignal and blue DAPI staining in nucleus. Arrows indicate green and red immunosignals overlapping in tanycytes from the central canal (A), cells of the area postrema (B) and median eminence (C). Central canal (CC), area postrema (AP), median eminence (ME). Scale bars = 20 µm.

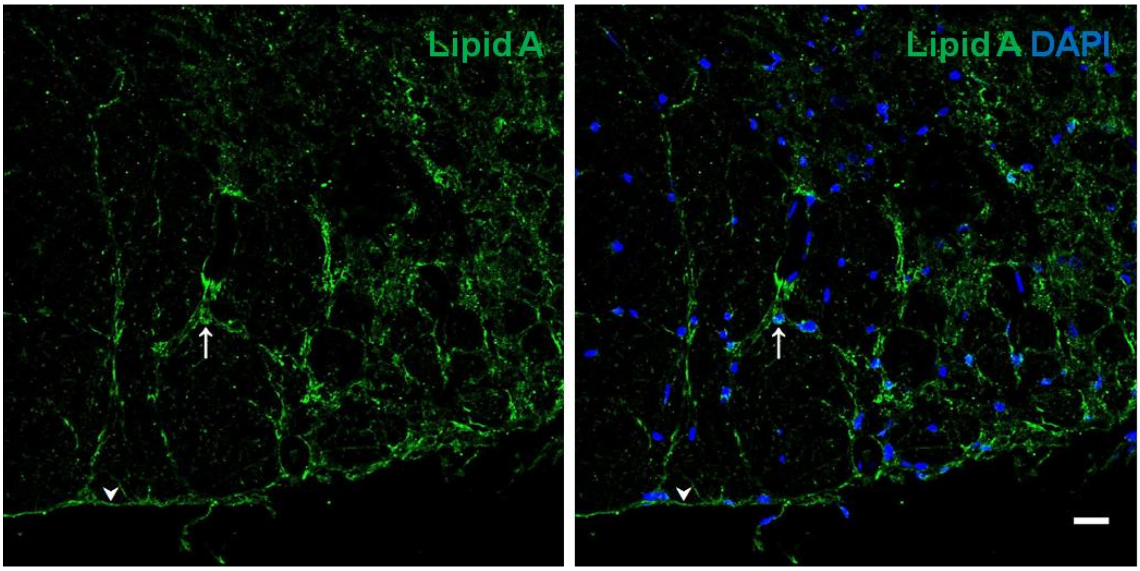

Figure S2. Lipid A can be detected in brain structures by different commercial antibodies. Immunoreactivity for lipid A using ab8467, Abcam antibody in medulla oblongata. Green corresponds to lipid A immunosignal, and blue DAPI staining in nucleus. Arrows indicate lipid A immunosignal in ramified cells. Head arrows indicate lipid A immunosignal in leptomeninges. Scale bar = 20 µm.

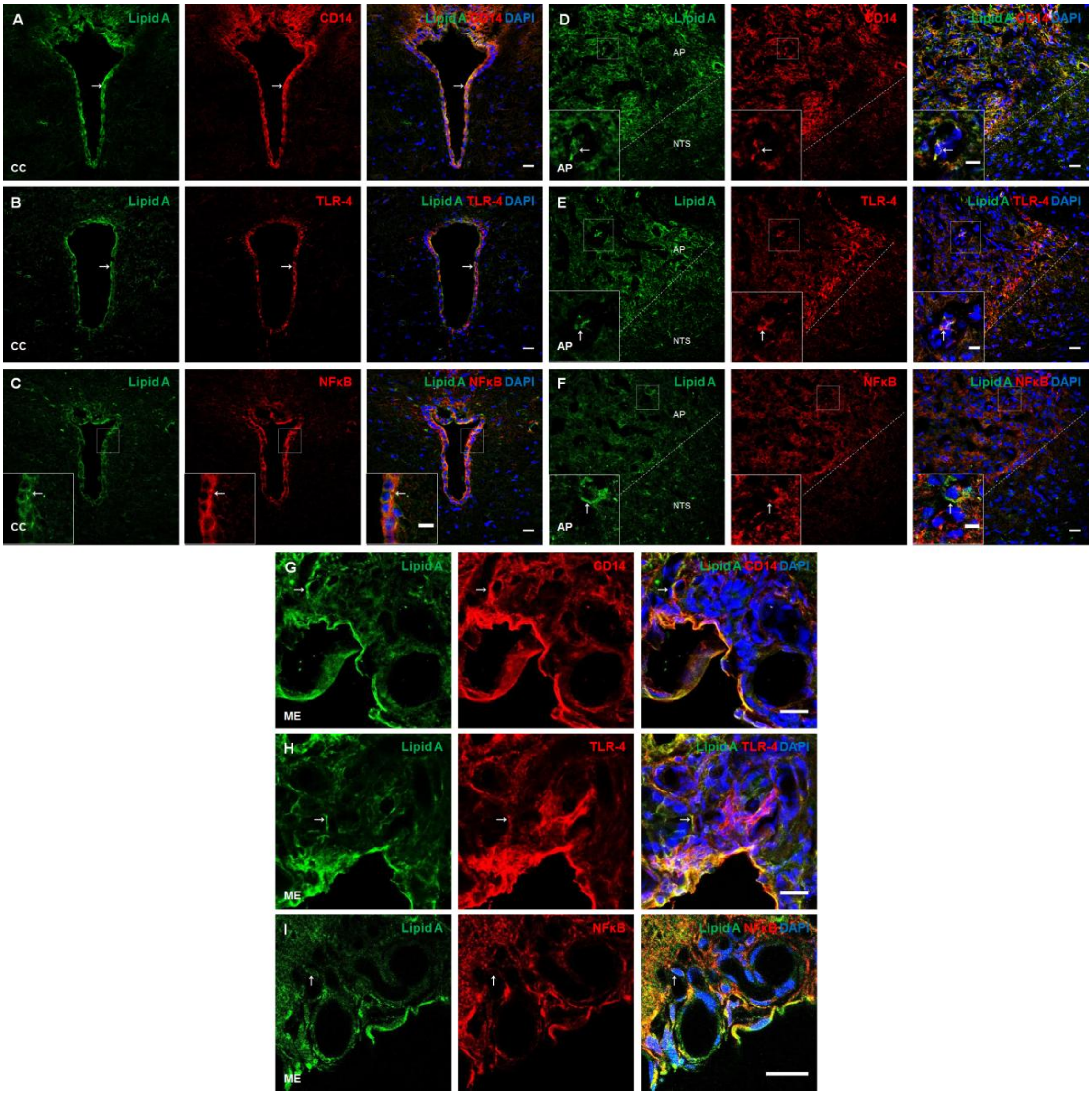

Figure S3. Lipid A co-localized with elements of the TLR-4 signaling pathway in tanycytes-like cells from central canal and circumventricular organs. Double immunofluorescence of Lipid A and CD14, TLR-4 or NFκB in the Central canal (A-C), area postrema (D-F) and median eminence (G-I). In all cases green corresponds to Lipid A immunosignal and blue DAPI staining in nucleus. Red immunosignal corresponds to CD14 (A, D and G), TLR-4 (B, E and H) and NFκB (C, F and I). Arrows indicate green and red immunosignals overlapping in, tanycytes from the central canal (A-C), and in cells of the area postrema (D-F) and median eminence (G-I). Dashed lines in D-F panels indicate the separation among AP and NTS. Central canal (CC), area postrema (AP), median eminence (ME), nucleus tractus solitarium (NTS). Scale bars = 20 µm, 10 µm for inserts.

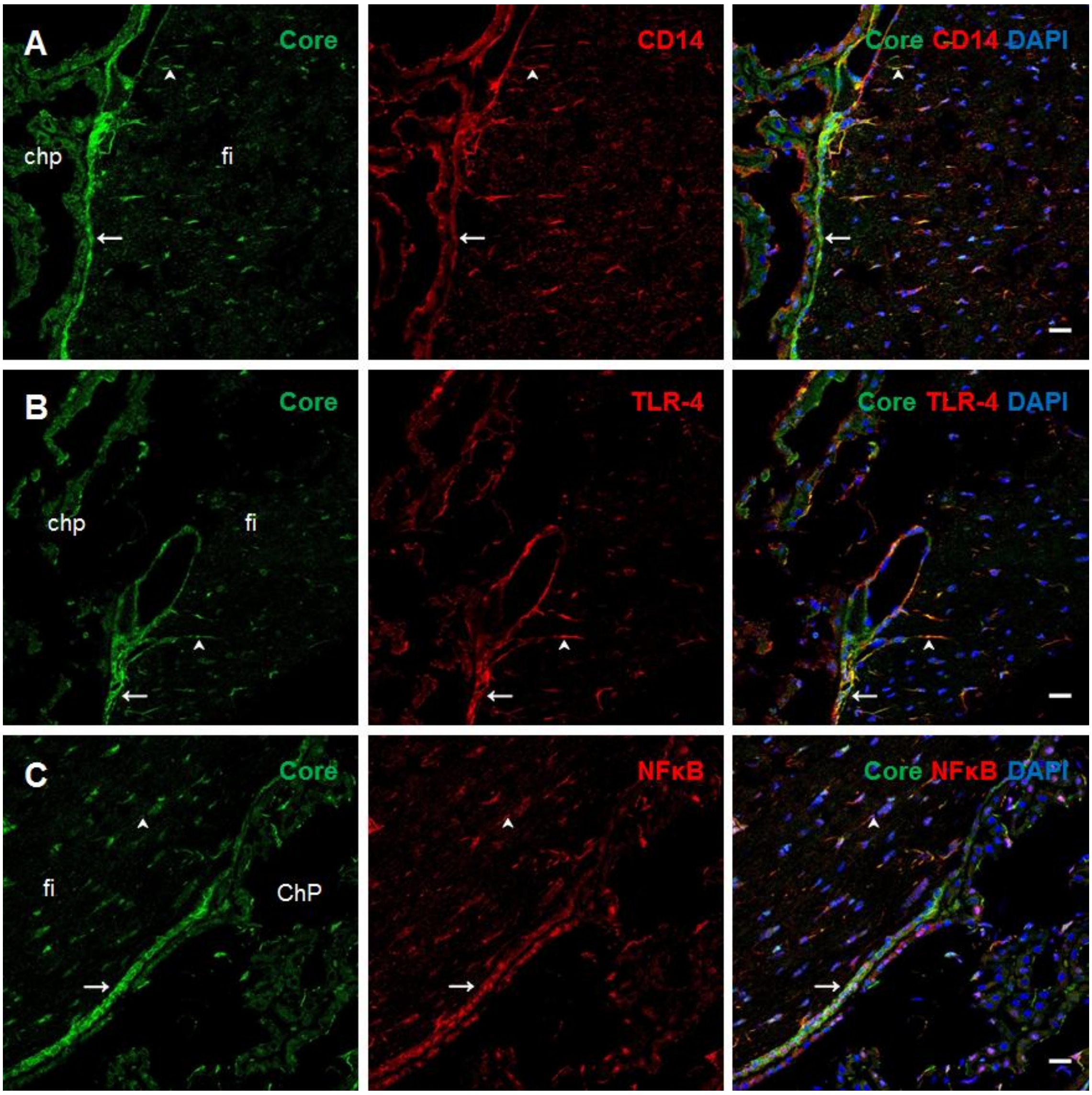

Figure S4. Core LPS co-localized with TLR-4 signaling pathway elements. Double immunofluorescences of Core and CD14, TLR-4 or NFκB in the lateral ventricle. In all cases green corresponds to core LPS region immunosignal and blue DAPI staining in nucleus. Red immunosignal corresponds to CD14 (A), TLR-4 (B), NFκB (C). Arrows indicate green and red immunosignals overlapping in tanycyte-like cells from the ventricle walls and head arrows indicate astrocyte-like cells in the hippocampal fissure (A-C). Choroid plexus (chp) and hippocampal fissure (fi). Scale bars = 20 µm

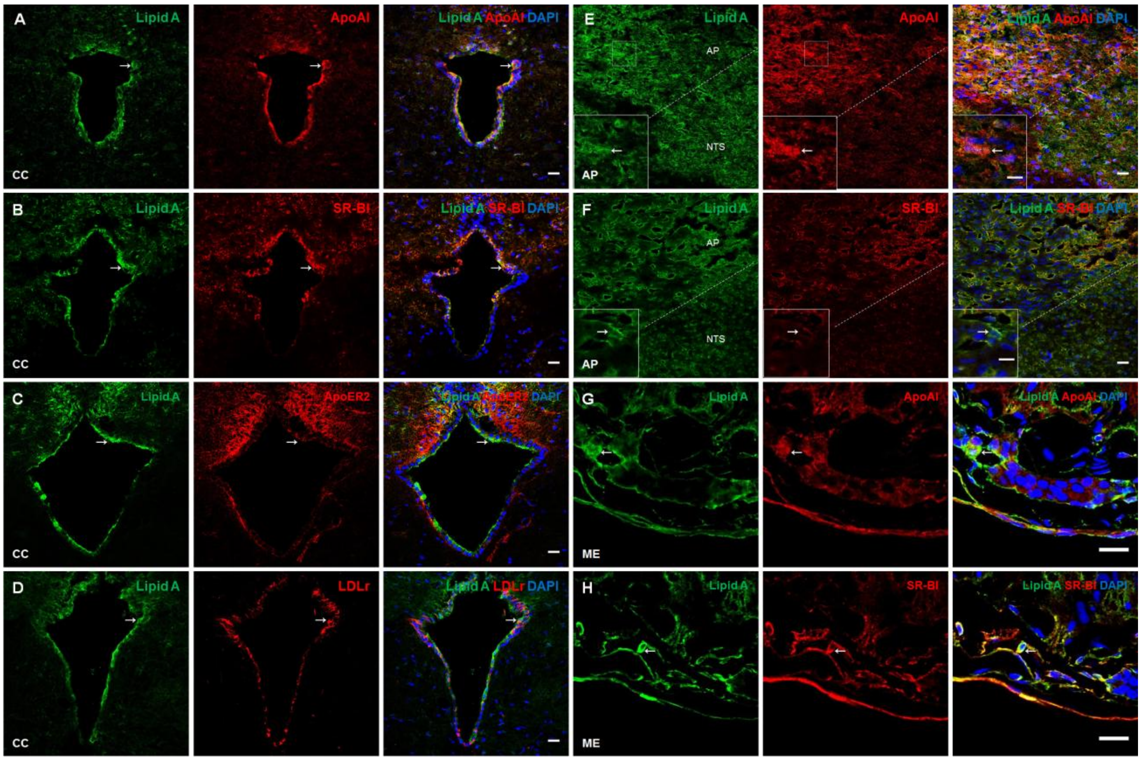

Figure S5. Lipid A co-localized with lipoproteins and their receptors in the central canal and circumventricular organs. Double immunofluorescence of lipid A and ApoAI, SR-BI, ApoER2 or LDLr in the central canal (A-D), area postrema (E-F), median eminence (G-H). In all cases green corresponds to Lipid A immunosignal and blue DAPI staining in nucleus. Red immunosignal corresponds to ApoAI (A, E and G), SR-BI (B, F and H) and ApoER2 (C) LDLr (D). Arrows indicate green and red immunosignals overlapping in, tanycyte-like cells from the central canal (A-D), and in cells of the area postrema (E-F) and median eminence (G-H). Dashed lines in D-F panels indicate the separation among AP and NTS. Central canal (CC), area postrema (AP), median eminence (ME), nucleus tractus solitarium (NTS). Scale bars = 20 µm, 10 µm for inserts.

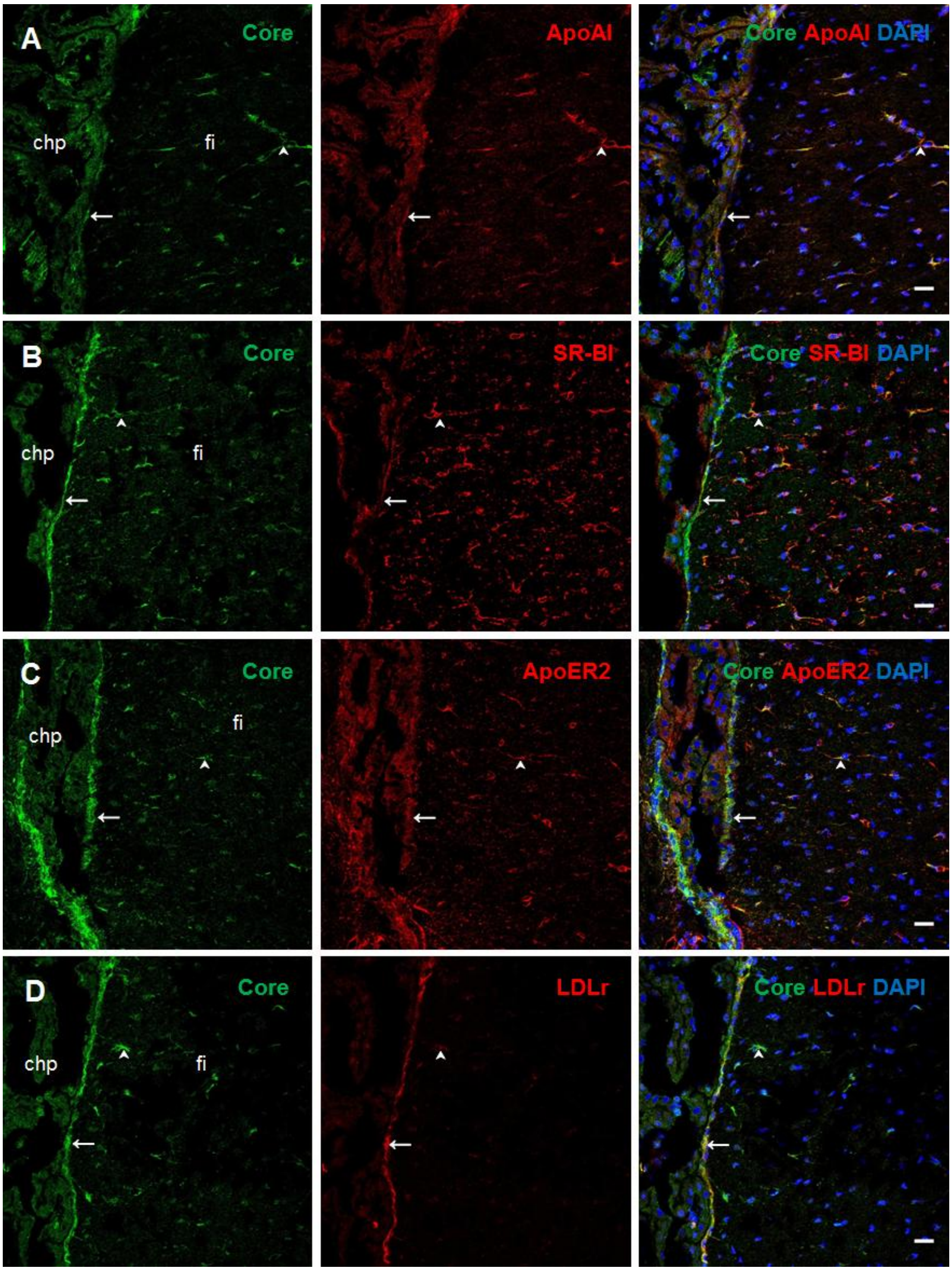

Figure S6. Core LPS region co-localized with lipoproteins and their receptors in the lateral ventricle. Double immunofluorescences of Core and ApoAI, SR-BI, ApoER2 or LDLr (A-D). In all cases green corresponds to core LPS region immunosignal and blue DAPI staining in nucleus. Red immunosignal corresponds, respectively, to ApoAI (A), SR-BI (B), ApoER2 (C) and LDLr (D). Arrows indicate green and red immunosignals overlapping in tanycytes from the ventricle walls and head arrows indicate astrocytes in the hippocampal fissure (A-D). Choroid plexus (chp) and hippocampal fissure (fi). Scale bars = 20 µm.

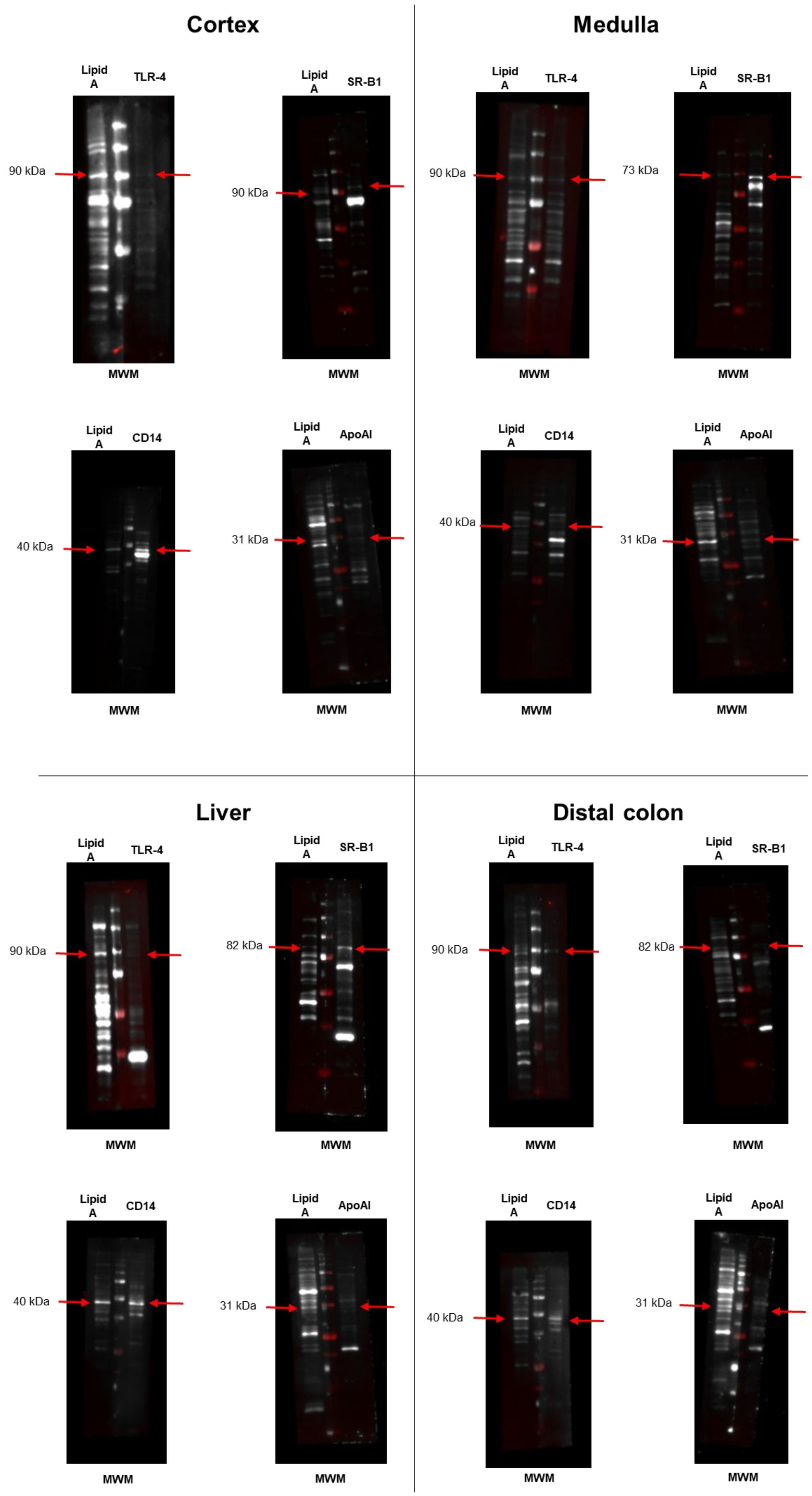
Cortex
Lipid A
TLR-4
90 kDa
MWM
Lipid A
SR-B1
90 kDa
MWM
Lipid A
CD14
40 kDa
MWM
Lipid A
ApoAI
31 kDa
MWM
Medulla
Lipid A
TLR-4
90 kDa
MWM
Lipid A
SR-B1
73 kDa
MWM
Lipid A
CD14
40 kDa
MWM
Lipid A
ApoAI
31 kDa
MWM
Liver
Lipid A
TLR-4
90 kDa
MWM
Lipid A
SR-B1
82 kDa
MWM
Lipid A
CD14
40 kDa
MWM
Lipid A
ApoAI
31 kDa
MWM
Distal colon
Lipid A
TLR-4
90 kDa
MWM
Lipid A
SR-B1
82 kDa
MWM
Lipid A
CD14
40 kDa
MWM
Lipid A
ApoAI
31 kDa
MWM

Figure S7. Original immunoblots of the comparative analysis for blots with similar MW between LPS and TLR-4, SR-B1, CD14, ApoAI detection in tissue homogenates. Simultaneous western blot analysis for lipid A and CD14, TLR-4 ApoAI or SR-B1 detection in cortex, medulla, liver and distal colon tissue homogenates from rats under control conditions. MWM: molecular weight marker. Red arrows indicate blots of similar MW for lipid A and target protein (CD14, TLR-4 ApoAI or SR-B1). Each c image corresponds to different simultaneous Western blot analysis, individually made for each lipid A and target protein comparison.

Table S1. Primary antibodies. (IF) Immunofluorescence, (WB) Western blot.

Marker	Antibody species	Company	Catalogue number	Dilution IF	Dilution WB
Lipid A	Goat	Acris	BP2235	1:50	1:500
Core	Mouse	HycultBiotech	HM6011	1:25	1:250
CD14	Rabbit	Abcam	ab203294	1:350	1:1000
TLR4	Rabbit	Stress marq	SPC-200D	1:350	1:750
NFκB p65	Rabbit	Cell signaling	8242s	1:200	
GFAP	Mouse	Abcam	ab10062	1:1000	
RECA-1	Mouse	abD serotec	MCA970R	1:1000	
LBP	Goat	Santa Cruz Biotech	sc-14666	1: 200	
ApoER2	Rabbit	LS Bio	LS-B5784	1:200	
LDLr	Rabbit	Abcam	ab30532	1:400	
ApoAI	Rabbit	Abcam	ab20453	1:50	1:4000
SR-BI	Rabbit	Novus Biologicals	NB400-104	1:1000	1:1000

Table S2. Secondary antibodies

**Conjugate**	**Antibody species**	**Host**	**Company**	**Reference**	**Dilution**
AlexaFluor®405	IgG Mouse	Goat	Life Technologies	A31553	1:50
AlexaFluor®488	IgG Goat	Donkey	Life Technologies	A11055	1:1000
AlexaFluor®488	IgG Rabbit	Donkey	Life Technologies	A21206	1:1000
AlexaFluor®488	IgG2a Mouse	Goat	Life Technologies	A21131	1:250
AlexaFluor®555	IgG Rabbit	Donkey	Life Technologies	A31572	1:1000
AlexaFluor®555	IgG Mouse	Donkey	Life Technologies	A31570	1:1000
AlexaFluor®555	IgG Goat	Donkey	Life Technologies	A21432	1:1000
AlexaFluor®647	IgG Rabbit	Donkey	Life Technologies	A31573	1:400
